# Human induced pluripotent stem cell engineering establishes a humanized mouse platform for pediatric low-grade glioma modeling

**DOI:** 10.1186/s40478-022-01428-2

**Published:** 2022-08-19

**Authors:** Corina Anastasaki, Jit Chatterjee, Olivia Cobb, Shilpa Sanapala, Suzanne M. Scheaffer, Amanda De Andrade Costa, Anna F. Wilson, Chloe M. Kernan, Ameera H. Zafar, Xia Ge, Joel R. Garbow, Fausto J. Rodriguez, David H. Gutmann

**Affiliations:** 1 Department of Neurology, Washington University School of Medicine, 660 S. Euclid Avenue, Box 8111, St. Louis, MO 63110 USA; 2 Department of Radiology, Washington University School of Medicine, St. Louis, MO 63110 USA; 3 Department of Pathology, David Geffen School of Medicine at UCLA, Los Angeles, CA 90095 USA

**Keywords:** Low-grade glioma, Pediatric brain tumor, Pilocytic astrocytoma, NF1, BRAF, Human induced pluripotent stem cells

## Abstract

A major obstacle to identifying improved treatments for pediatric low-grade brain tumors (gliomas) is the inability to reproducibly generate human xenografts. To surmount this barrier, we leveraged human induced pluripotent stem cell (hiPSC) engineering to generate low-grade gliomas (LGGs) harboring the two most common pediatric pilocytic astrocytoma-associated molecular alterations, *NF1* loss and *KIAA1549:BRAF* fusion. Herein, we identified that hiPSC-derived neuroglial progenitor populations (neural progenitors, glial restricted progenitors and oligodendrocyte progenitors), but not terminally differentiated astrocytes, give rise to tumors retaining LGG histologic features for at least 6 months in vivo. Additionally, we demonstrated that hiPSC-LGG xenograft formation requires the absence of CD4 T cell-mediated induction of astrocytic Cxcl10 expression. Genetic *Cxcl10* ablation is both necessary and sufficient for human LGG xenograft development, which additionally enables the successful long-term growth of patient-derived pediatric LGGs in vivo. Lastly, MEK inhibitor (PD0325901) treatment increased hiPSC-LGG cell apoptosis and reduced proliferation both in vitro and in vivo. Collectively, this study establishes a tractable experimental humanized platform to elucidate the pathogenesis of and potential therapeutic opportunities for childhood brain tumors.

**Supplementary Information:**

The online version contains supplementary material available at 10.1186/s40478-022-01428-2.

## Introduction

Low-grade gliomas (LGGs; World Health Organization grade 1 and 2 astrocytomas) are the most frequently occurring cancers of the central nervous system (CNS) in children [41], accounting for one third of all pediatric brain tumors [44]. The most common pediatric LGG is the grade 1 pilocytic astrocytoma (PA), which arises sporadically or in the context of the neurofibromatosis type 1 (NF1) tumor predisposition syndrome. In contrast to gliomas in adults, pediatric LGGs are typically slow-growing neoplasms located in the cerebellum, brainstem, and optic pathway [61], with an overall 10-year survival rate of > 90% [19]. Importantly, PAs in children rarely progress to high-grade malignancy and infrequently result in death. As such, pediatric LGGs represent a chronic condition that causes significant life-long morbidity [5], including vision loss [16], neurologic deficits, and endocrine complications [18, 54].

While preclinical mouse and pig models of NF1-associated optic pathway glioma (NF1-OPG [7, 29, 70]) and *BRAF*-driven [20] sporadic pediatric LGGs have helped elucidate the pathobiology of these tumors and served as platforms for therapeutic drug testing [22, 34], they only partially capture the essential properties of their human counterparts. Unfortunately, efforts to develop patient-derived pediatric LGG xenograft (PDX) models have been hindered by multiple factors, including their intrinsic slow growth rates [28], propensity to undergo premature senescence [30, 49], low clonogenic frequency [58], and heavy dependence on a supportive microenvironment [47]. In addition, the few existing human pediatric LGG xenograft models harbor genetic mutations (*TP53/CDKN1A* or *CDKN2A/RB1* alterations) uncharacteristic of these childhood gliomas, especially PAs, which were specifically introduced to permit pediatric LGG cells to escape cellular senescence [55, 62]. In the current report, we leverage human induced pluripotent stem cell (hiPSC) engineering to generate humanized NF1-associated and sporadic *KIAA1549:BRAF*-driven pediatric LGG models, identify potential cells of origin for these tumors, and discover a CD4^+^ T cell-astrocyte Cxcl10 axis critical for LGG xenograft formation. Disruption of Cxcl10 expression established a new murine platform for in vivo patient-derived pediatric LGG xenograft modeling and therapeutic evaluation.

## Results

### *NF1*-null and *KIAA1549:BRAF*-expressing human induced pluripotent stem cell (hiPSC)-derived neural progenitor cells (iNPCs) exhibit increased cell proliferation

To model *NF1* loss of heterozygosity in pediatric LGGs arising in the NF1 brain tumor predisposition syndrome, we engineered two different hiPSCs lines, each with a distinct homozygous germline NF1 patient-derived *NF1* mutation (c.2041C > T^−/−^ and c.6513T > A^−/−^; Additional file 1: Fig. S1a). Although the cell of origin for human pediatric LGGs is currently unknown, murine *Nf1* LGGs of the optic nerve/chiasm (optic pathway gliomas; OPGs) arise from neural stem or neuroglial (progenitor) cells [36]. As such, we first differentiated *NF1-*null (c.2041C > T^−/−^ and c.6513T > A^−/−^) and control (isogenic iPSCs that have undergone identical CRISPR/Cas9 processing as the *NF1*-null iPSC lines, but without the introduction of a mutation) hiPSCs into multipotent human neural stem cells (iNPCs) capable of generating both neuronal and glial lineage cells (Additional file 1: Fig. S1a). Consistent with the established role of the *NF1* protein, neurofibromin, as a negative RAS regulator [43], these *NF1-*null iNPCs had increased RAS activity (2041C > T^−/−^, 9.2-fold; 6513T > A^−/−^, 13.9-fold; 2041C > T^+/−^, 2.2-fold^−^; 6513T > A^+/−^, 1.8-fold relative to CTL iNPCs; Additional file 1: Fig. S1b), but similar cAMP levels (2041C > T^−/−^, 51% reduction; 6513T > A^−/−^, 57% reduction; 2041C > T^+/−^, 49% reduction; 6513T > A^+/−^, 61% reduction; Additional file 1: Fig. S1c), relative to *NF1-*mutant iNPCs heterozygous for the same germline *NF1* mutations, as we previously reported in mice [4]. In addition, to model sporadic pediatric LGGs resulting from genomic rearrangements involving the *BRAF* kinase gene [32, 67], we generated *KIAA1549:BRAF*-expressing iNPCs. Both *NF1-*null and *KIAA1549:BRAF*-expressing iNPCs had increased proliferation, as measured by BrdU incorporation (2041C > T^−/−^, 7.9-fold; 6513T > A^−/−^, 8.2-fold; 2041C > T^+/−^, 3.3-fold^−^; 6513T > A^+/−^, 3.5-fold; *KIAA1549:BRAF*, 7.5-fold) and direct cell counting (2041C > T^−/−^, 4.2-fold; 6513T > A^−/−^, 4.6-fold; 2041C > T^+/−^, 1.8-fold^−^; 6513T > A^+/−^, 2-fold; *KIAA1549:BRAF*, 3.1-fold), relative to control iNPCs (Additional file 1: Fig. S1d, e). Moreover, *KIAA1549:BRAF*-expressing iNPCs demonstrated increased phosphorylation of ERK1/2 relative to their isogenic controls, indicative of increased MAPK pathway activation (Additional file 1: Fig. S1f).

### iNPCs form LGGs in ***Rag1***^−/−^ mice

**Fig. 1:**
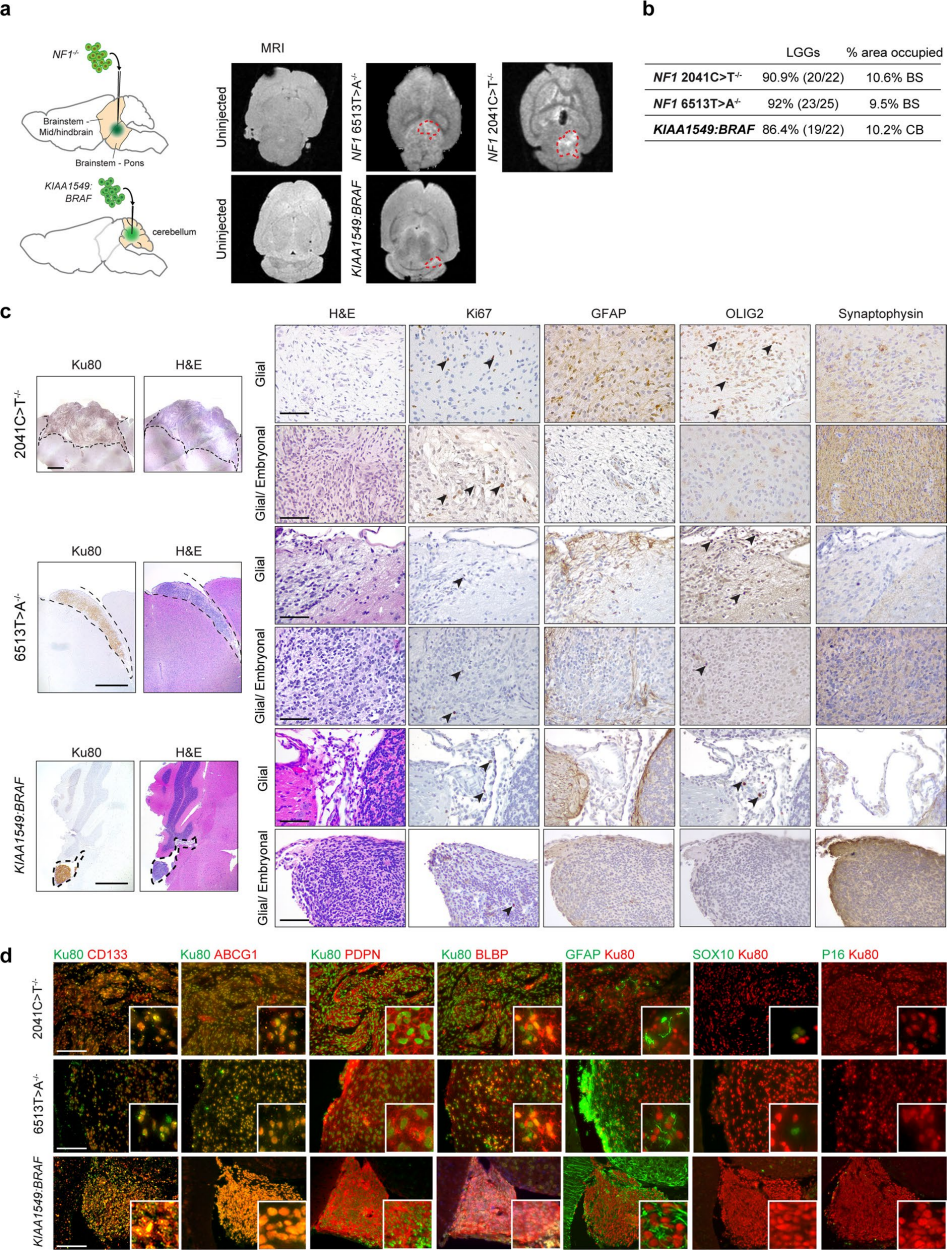
*Rag1*^*−/−*^ mice develop *NF1-*null and *KIAA1549:BRAF*-expressing low-grade gliomas (LGGs) **a** Injection of *NF1*-null iNPCs (2041C > T^−/−^, 6513T > A^−/−^) into the brainstems or *KIAA1549:BRAF*-expressing iNPCs into the cerebella of *Rag1*^*−/−*^ mice result in the formation of LGGs detectable by MRI (denoted by red dotted lines). **b** Summary of injected *Rag1*^*−/−*^ mice harboring iNPC LGGs. **c** LGGs are hypercellular (H&E staining) and immunopositive for Ku80 (human-specific antibody), Ki67, glial (GFAP, OLIG2) and neuronal (synaptophysin) marker expression, by 1 month post-injection (mpi). The tumors had both glial only (top panels) and mixed embryonal/glial (lower panels) histological characteristics. Scale bars: left, Ku80/ H&E panels, 1 mm; other panels, 100 µm. **d** LGGs contain CD133^+^, ABCG1^+^, PDPN^+^, BLBP^+^ and GFAP^+^ cells (white arrowheads). No Ku80^+^ LGG tumor cells had SOX10 or p16 expression. Scale bar, 100 µm

To determine whether *NF1-*null iNPCs generate LGGs in immunocompromised mice, 1 × 10^4^ to 5 × 10^5^ CTL or *NF1-*null iNPCs were implanted into the brainstems of *Rag1*^−/−^ mice at 0–3 days of age (PN0-3). We specifically chose the brainstem for several reasons: (1) it is the second most common brain location for pediatric LGGs arising in children with NF1 [21, 42], (2) we have previously used brainstem injections for murine *Nf1*-OPG stem cell tumor modeling [47], and (3) injections into the mouse optic nerve, the most common site for NF1-pediatric LGGs, create major tissue damage and induce a reactive immune microenvironment. Using established neuropathological criteria for pediatric LGGs [10], we defined a LGG as (1) a mass-occupying lesion with architectural distortion by standard H&E staining (and by MRI in a subset of cases), with (2) increased proliferation (Ki67 labeling index > 1%) and (3) immunopositivity for glial immunohistochemical markers used in the routine diagnosis of human low-grade gliomas (e.g., GFAP and OLIG2). Based on these criteria and leveraging this hiPSC/murine platform, both *NF1-*null iNPC lines (2041C > T^−/−^, 6513T > A^−/−^) formed LGGs 1 month post-injection (mpi) in approximately 50% of mice injected with 1 × 10^5^ iNPCs and in > 85% of animals injected with 5 × 10^5^ iNPCs (Additional file 1: Fig. S2a). Similarly, to model sporadic pediatric LGGs, we orthotopically transplanted 5 × 10^5^ *KIAA1549:BRAF*-expressing iNPCs into the cerebella of *Rag1*^−/−^ mice, the most common location for sporadic PA with this molecular alteration. Over 85% of all *Rag1*^−/−^ mice injected with *KIAA1549:BRAF*-expressing iNPCs formed LGGs at 1mpi (Fig. 1a, b). LGG sections were reviewed by an expert human neuropathologist (F.J.R.).

NF1-associated and *KIAA1549:BRAF*-driven lesions were also detectable by magnetic resonance imaging (4.7-Tesla MRI, Fig. 1a) and exhibited many of the histopathologic features of human pediatric LGGs. These tumors were composed of human cells (Ku80^+^ cells), were hypercellular, mostly parenchymal with exophytic components, either anterior or lateral to the midbrain/brainstem tissue (*NF1-*null iNPC tumors) or anterior to the cerebellum (*KIAA1549:BRAF-*expressing iNPC tumors), and well-circumscribed (Fig. 1c). The tumors contained GFAP- and OLIG2-immunopositive cells (Fig. 1c), as seen in pediatric LGGs. All of the iNPC-lesions contained both glial areas, as determined by H&E, GFAP and OLIG2 (glial) immunopositivity, and embryonal-like hypercellular areas with neuronal (synaptophysin^+^) components, some of which contained neuroepithelial rosettes. Since the resulting tumors did not exhibit some histologic features routinely observed in pilocytic astrocytomas (eosinophilic granular bodies, Rosenthal fibers), they were classified as LGGs, which is the routine diagnostic approach in clinical practice. Moreover, the Ku80^+^ cells in these LGGs also expressed CD133 and ABCG1 (Fig. 1d), markers of glioma stem cells [15, 59], but were immunonegative for SOX10 and p16 expression, which can be observed in some patient pediatric LGGs (Fig. 1d). iNPC LGGs were negative for the OCT4 and NANOG hiPSC pluripotency markers, as well as for SMA and AFP endoderm and mesoderm markers, but were immunopositive for the Nestin neural stem cell marker (Additional file 1: Fig. S2b). Additionally, *KIAA1549:BRAF*-expressing LGGs demonstrated increased ERK1/2 activity relative to the surrounding non-neoplastic brain tissue (Additional file 1: Fig. S2c). Importantly, neither heterozygous *NF1*-mutant nor control iNPCs formed LGGs in *Rag1*^−/−^ mice (Additional file 1: Fig. S2d), consistent with the requirement for *NF1* loss of heterozygosity in NF1-LGG tumorigenesis.

RNA sequencing or methylation studies were not used to compare the humanized iNPC-LGGs with resected patient LGGs: iNPC-LGGs are composed entirely of human neoplastic cells and cannot be directly compared with human LGG biospecimens, in which 30–50% of the cells are monocytes, neurons, and T cells [13, 23, 50, 51, 57]. In addition, while methylation has proven valuable for separating classes of brain tumors, it is not as accurate in distinguishing subclasses of pediatric LGGs and glioneuronal tumors. This is due in part to the variable contributions of non-neoplastic elements, with the diagnostic standard involving histology, immunohistochemistry, and genetic analysis for the specific drivers involved (e.g., *BRAF*, *NF1*) [12, 52]. For these reasons, we employed two additional complementary approaches to compare hiPSC-LGGs to their spontaneously arising clinical counterparts. First, leveraging human bulk RNA sequencing data [46], we identified *PDPN* as a gene differentially expressed both in NF1-associated and sporadic pilocytic astrocytomas relative to control non-neoplastic brain tissue (Additional file 1: Fig. S2e). Second, we found that *FABP7,* which we previously showed to be overexpressed in mouse optic gliomas relative to control optic nerve by bulk RNA sequencing [15], was increased both in NF1-associated and sporadic pediatric LGGs relative to control non-neoplastic human brain (Additional file 1: Fig. S2e). Similar to their spontaneously arising pediatric LGG counterparts, both PDPN and BLBP (*FABP7* protein) were expressed in the humanized *NF1-*null and *KIAA1549:BRAF-*associated LGGs in situ (Fig. 1d).

**Fig. 2:**
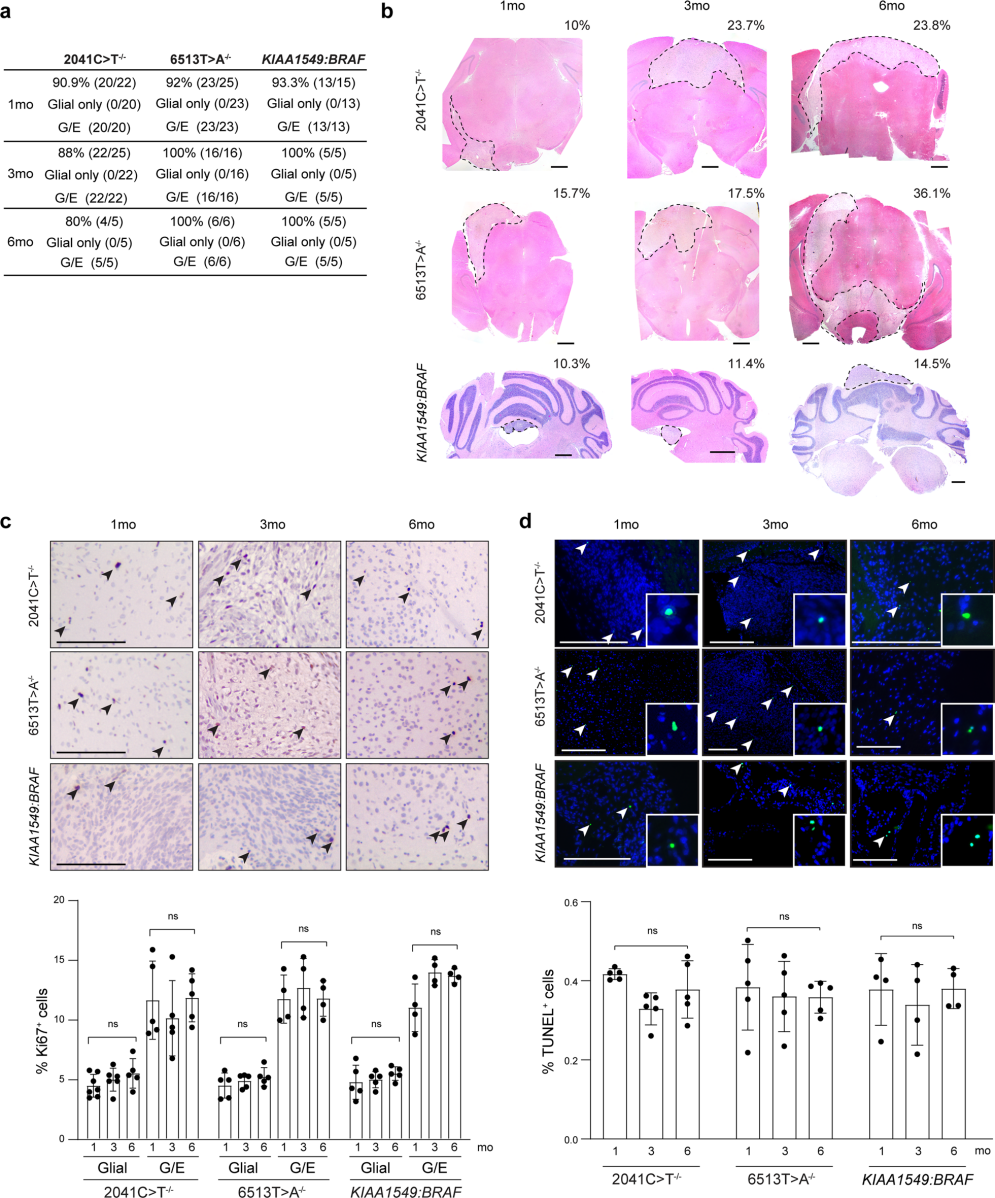
*Rag1*^*−/−*^ mice develop persistent LGGs **a** Percentage of *Rag1*^−/−^ mice harboring LGGs at 1, 3 and 6 mpi. The total number of injected mice is denoted in the parentheses. G, glial only; G/E, mixed glial/embryonal. **b** Representative H&E staining of *NF1-*null and *KIAA1549:BRAF*-expressing LGGs 1, 3 and 6 mpi. The lesions are outlined by the dotted lines. The percentage of the area occupied by the LGG is indicated within each panel. Scale bars, 200 µm. **c** Representative Ki67 immunohistochemistry images (top panel) and percentages of Ki67^+^ cells in *Rag1*^*−/−*^ mice harboring *NF1-*null or *KIAA1549:BRAF*-expressing iNPC glial or mixed/glial embryonal tumors at 1, 3 and 6 mpi. Scale bars, 200 µm **d** No change in TUNEL^+^ cells (apoptotic cells) in *NF1-*null and *KIAA1549:BRAF*-expressing iNPC-derived LGGs were observed 1 (0.41%; 0.38%; 0.38%), 3 (0.36%, 0.37%; 0.34%), and 6 months (0.38%, 0.37%; 0.38%) after injection. Scale bars, 100 µm. Data are represented as means ± SEM, one-way ANOVA with Bonferroni post-test correction, Individual *p* values are indicated within each graph. *ns* not significant

As expected for pediatric low-grade tumors, and mirroring clinical observations, mice with hiPSC-derived LGGs did not exhibit increased mortality nor obvious abnormal neurologic findings. To determine whether these lesions reflected the chronic non-malignant nature of pediatric LGGs, we assessed tumors at 1, 3 and 6 mpi (Fig. 2). Using power calculations with a two-sided, two portions test, where power was set at 80% and significance at 0.05, we estimated that a minimum of 4, 4, and 5 mice would be required to detect tumor formation at 1 mpi, 3 mpi and 6 mpi, respectively. With appropriately powered cohorts, mice injected with *NF1-*null and *KIAA1549:BRAF-*expressing iNPCs formed LGGs that were histologically similar at 1, 3 and 6 mpi (Fig. 2a). While these lesions grew in size over time, occupying on average 10–36% of the brainstem or 10–15% of the cerebellum, respectively (Fig. 2b), they had similar proliferative indices (Ki67^+^ cells) at 1, 3 and 6 mpi (Fig. 2c). The glioma tumor areas exhibited low proliferative indices (4–6% Ki67^+^ cells), within the upper range of proliferation rates seen in pediatric PAs [64], whereas the embryonal-like areas exhibited slightly higher proliferative rates (8–15% Ki67^+^ cells) (Fig. 2a, c). There was no evidence of increased apoptosis (0.38–0.41% TUNEL^+^ cells; Fig. 2d) or increased cellular senescence (0.3–0.4% β-galactosidase^+^ cells; Additional file 1: Fig. S2f) in vivo. Taken together, these findings demonstrate that iNPCs generate both NF1-associated and sporadic LGGs in vivo.

### hiPSC-derived glial restricted progenitors (iGRPs) and oligodendrocyte progenitors (iOPCs) form LGGs in ***Rag1***^−/−^ mice

**Fig. 3:**
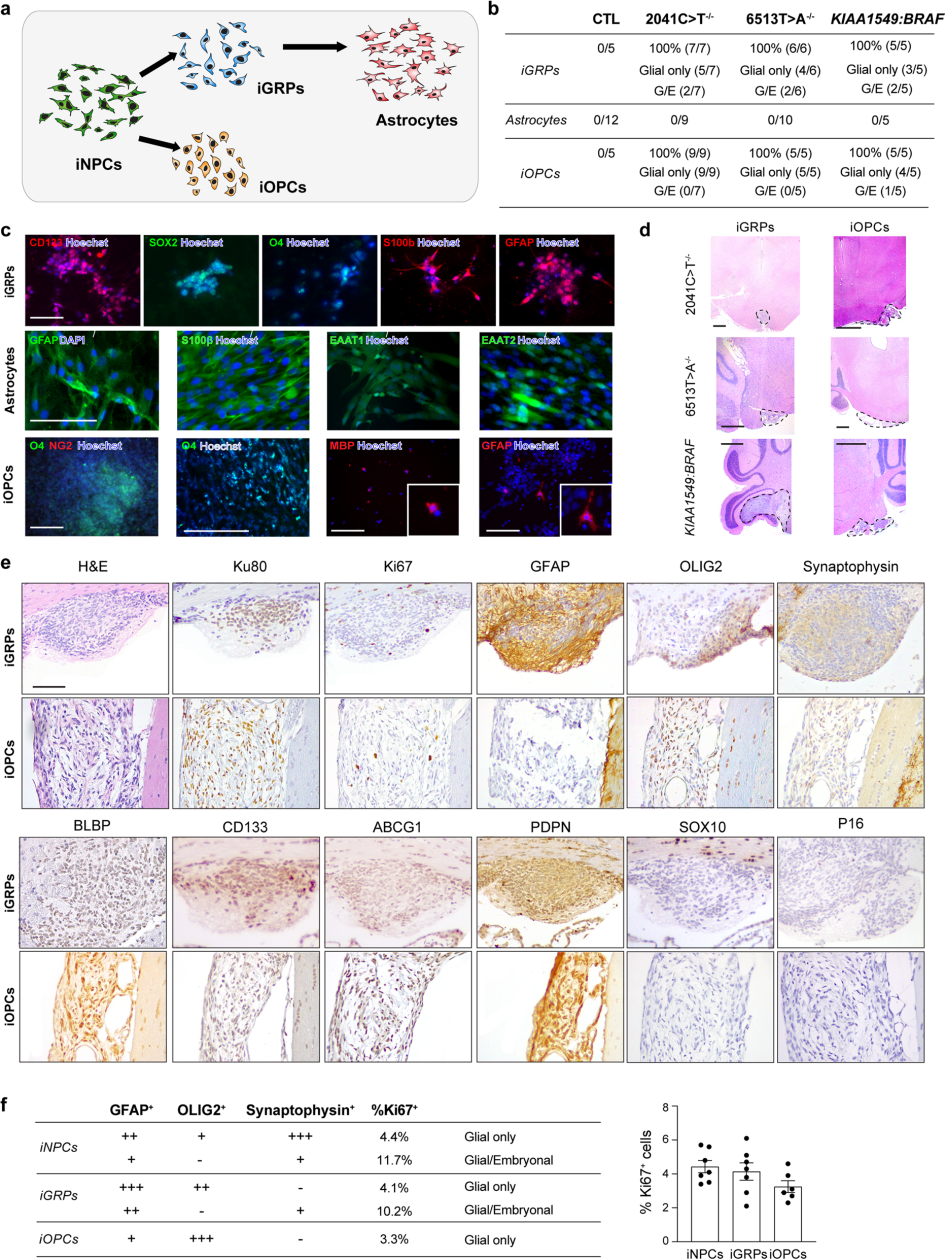
iGRPs form LGGs in *Rag1*^−/−^ mice **a** Differentiation of human iNPCs into iOPCs, iGRPs and astrocytes. **b** *Rag1*^*−/−*^ mice harbor LGGs at 1mpi following CTL, 2041C > T^−/−^ and 6513T > A^−/−^ *NF1*-null iGRP and iOPC brainstem injections, as well as *KIAA1549:BRAF*-expressing iGRP and iOPC cerebellum injections. No LGGs were observed following *NF1*-null or *KIAA1549:BRAF*-expressing hiPSC-astrocyte injections. G, glial only; G/E, mixed embryonal/glial. **c** Analysis of 2041C > T^−/−^ *NF1*-null differentiated cells revealed CD133^+^, SOX2^+^, ABCG1^+^, O4^+^, GFAP^+^ and S100β ^+^ iGRPs (top panel), GFAP^+^, S100b^+^, EAAT^+^ and EAAT2+ astrocytes (middle panel) and O4^+^, MBP^+^, GFAP^+^ and NG2^neg^ iOPCs ( lower panel). **d** Representative low-magnification H&E images of 2041C > T^−/−^ and 6513 T > A^−/−^ *NF1*-null and *KIAA1549:BRAF*-expressing iGRP and iOPC LGGs. Scale bars, 1 mm. **e** H&E, Ku80, Ki67, GFAP, OLIG2, synaptophysin, BLBP, CD133, ABCG1, PDPN, SOX10 and P16 immunostaining images of representative 2041C > T^−/−^ *NF1*-null iGRP- (top) and iOPC- (bottom) LGGs at 1mpi. Scale bars, 100 µm. (**f**) Summary of relative immunopositivity scoring for GFAP, OLIG2, synaptophysin and Ki67 in LGG-bearing 1mo mice. −, 0%; +, < 25%; ++, 50%; +++, > 75% immunopositive cells. Data are represented as means ± SEM, one-way ANOVA with Bonferroni post-test correction, *p* values are not significant

Because iNPCs are a multipotent population that generates neuronal, glial and oligodendroglial progenitor-like cells in vitro and within the LGGs in vivo (Fig. 1d), we leveraged this hiPSC platform to examine the potential of derivative restricted progenitors to serve as cells of origin for LGGs. To this end, we differentiated control, *NF1-*null, and *KIAA1549:BRAF*-expressing iNPCs into glial restricted progenitors (iGRPs: CD133^+^, SOX2^+^, O4^+^, GFAP^+^ and S100β^+^ cells), oligodendrocyte progenitor cells (iOPCs: O4^+^, MBP^+^, GFAP^+^ and NG2^neg^ cells), and astrocytes (GFAP^+^, S100β^+^, EAAT1^+^ and EAAT2^+^ cells) (Fig. 3a) for injection into the brainstems of *Rag1*^−/−^ mice (Additional file 1: Figs. S3 and S4). 1 month following the injection of 5 × 10^5^ *NF1-*null or *KIAA1549:BRAF*-expressing cells, transplanted astrocytes did not form tumors (Fig. 3b), suggesting that terminally-differentiated preneoplastic astrocytes are unlikely to be the cells of origin for pediatric LGGs, similar to that observed for murine *Nf1* optic gliomas [36]. In contrast, LGGs formed in 100% of *Rag1*^−/−^ mice injected with 5 × 10^5^ *NF1-*null (n = 13) and *KIAA1549:BRAF*-expressing (n = 5) iGRPs or 5 × 10^5^ *NF1-*null (n = 14) and *KIAA1549:BRAF*-expressing (n = 5) iOPCs (Fig. 3b, d, Additional file 1: Fig. S4).

While both iGRPs and iOPCs formed LGGs, they each exhibited unique histopathologic features seen in human PAs: iGRP LGGs were compact and hypercellular, forming as either purely parenchymal masses or parenchymal masses with exophytic components. A small subset of iGRP LGGs contained glial/embryonal tumor areas that were moderately immunopositive for synaptophysin (Fig. 3d, e, Additional file 1: Fig. S4). In contrast, iOPC LGGs had a looser, less compact architecture with numerous microcysts, and were located exophytically between the midbrain/brainstem and the hippocampus. In contrast to the strongly GFAP-immunoreactive iGRP LGGs, iOPC LGGs were more intensely OLIG2-immunoreactive and mostly negative for GFAP and synaptophysin expression (Fig. 3d, e, Additional file 1: Fig. S4). Both iGRP and iOPC-LGGs exhibited low proliferative indexes (4.1% and 3.2% Ki67^+^ cells, respectively) (Fig. 3e), similar to most childhood brainstem pediatric LGGs.

### iNPC-LGG formation requires CD4^+^ T cell depletion

**Fig. 4:**
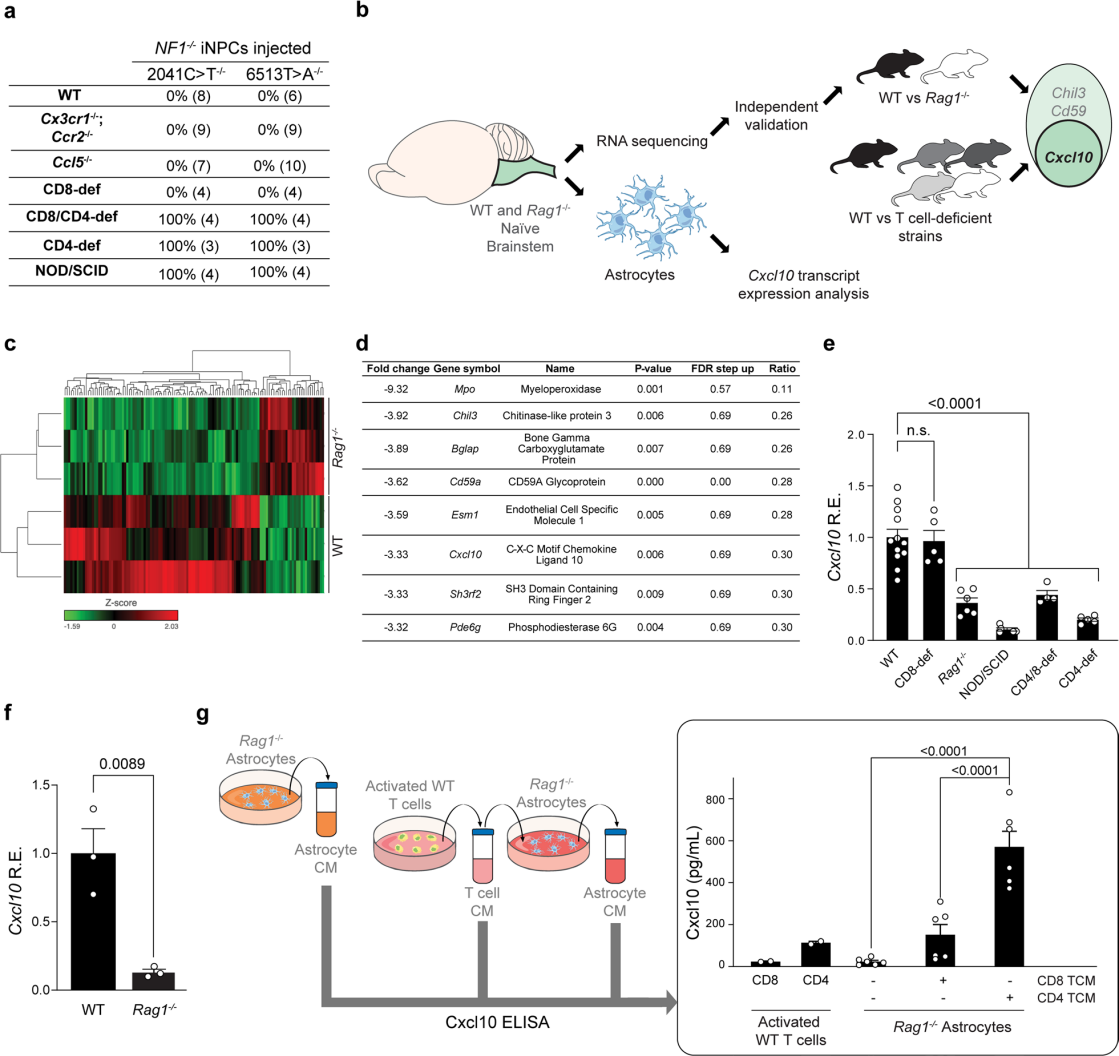
CD4^+^ T cells control iNPC LGG formation in a Cxcl10-dependent manner **a ** Mouse strains harboring *NF1-*null iNPC-derived LGGs at 1 mpi. **b** Schematic detailing the experimental design. **c** Heat map analysis of RNA sequencing performed on whole brainstem tissues from naïve wild type (WT) and *Rag1*^−/−^ mice reveals segregation of transcript expression. **d** List of > 3-fold differentially expressed transcripts from the brainstems of *Rag1*^−/−^ mice relative to WT controls. **e** Relative expression (R.E.) of *Cxcl10* transcripts in WT and immunodeficient (CD8-deficient*, Rag1*^*−/−*^, *NOD/SCID*, CD4/8-deficient and CD4-deficient) mice. n = 5; data are represented as means ± SEM; one-way ANOVA with Bonferroni post-test correction. Individual *p* values are indicated above each bar. ns, not significant. **f** Relative *Cxcl10* expression is reduced in *Rag1*^*−/−*^ astrocytes compared to WT controls. n = 3; data are represented as means ± SEM, two-tailed student’s t-test; *p* = 0.0089. **g** Schematic of the experimental design, and histogram demonstrating that Cxcl10 protein levels are increased by 6.5- and 24.4-fold in *Rag1*^−/−^ astrocytes treated with CD8^+^ and CD4^+^ T cell conditioned media (TCM), respectively, relative to untreated *Rag1*^*−/−*^ astrocytes. n = 6; one-way ANOVA with Bonferroni post-test correction; individual *p* values are indicated above each bar

Similar to human patient brain tumor xenografts, iNPC lineage cells did not form LGGs following injection into wild type mice (Fig. 4a). To identify mouse strains that permit LGG formation, *NF1-*null iNPCs were injected into the brainstems of a series of immunodefective mouse strains (listed in Fig. 4a). Whereas LGGs readily formed in *NOD/SCID,* CD4-deficient and CD4/CD8-deficient mice at 1mpi, no tumors developed in CD8-deficient mice or strains deficient in the expression of microglia or T cell chemokine receptors (*Cx3cr1*, *Ccr2*) [45], components required for murine *Nf1* optic glioma formation and growth (Additional file 1: Fig. S5a). To identify potential responsible molecular etiologies underlying CD4^+^ T cell deficiency-mediated LGG formation, we performed transcriptomal analysis on whole brainstems of wild type and *Rag1*^−/−^ mice (Fig. 4b, c; Additional file 1: Fig. S5b). We initially identified eight downregulated transcripts and one upregulated transcript in *Rag1*^−/−^ relative to wild type mouse brainstems (Fig. 4d). While three downregulated transcripts were validated in independently-acquired *Rag1*^−/−^ brainstem samples (*Chil3*, *Cd59*, *Cxcl10*; Fig. 4e; Additional file 1: Fig. S5c), only *Cxcl10* expression was decreased in all immunodefective mouse strains that permitted LGG formation.

Since Cxcl10, a cytokine belonging to the CXC chemokine family, is predominantly expressed by microglia and astrocytes [68], we next analyzed these cell types in uninjected wild type and immunodeficient mouse brainstems. Whereas microglia density and morphology was relatively unaltered in all uninjected mouse brains analyzed (Additional file 1: Fig. S6a), there were fewer GFAP^+^, EAAT2^+^ and ALDH1L1^+^ astrocytes in the brainstems of all immunodefective mouse strains that permitted LGG formation (Additional file 1: Fig. S6b). This finding suggested that LGG formation may require a deficit in astrocytes. Consistent with an astrocyte defect, astrocytes isolated from *Rag1*^*−/−*^ mice had reduced *Cxcl10* expression relative to wild type controls (86.3% reduction, Fig. 4f). Importantly, incubation of *Rag1*^−/−^ astrocytes with activated wild type mouse T cell-conditioned medium (TCM) induced Cxcl10 protein production. The greatest increase in Cxcl10 protein production was observed after induction of *Rag1*^−/−^ astrocytes with TCM from CD4^+^ (24.4-fold), relative to CD8^+^ (6.5-fold), T cells (Fig. 4g). Taken together, these results indicate that reduced astrocytic *Cxcl10* expression in glioma-bearing mouse strains likely reflects an absence of CD4 T cells, which induce Cxcl10 expression in astrocytes to hinder LGG growth in vivo.

### Cxcl10 inhibits pediatric LGG formation

**Fig. 5:**
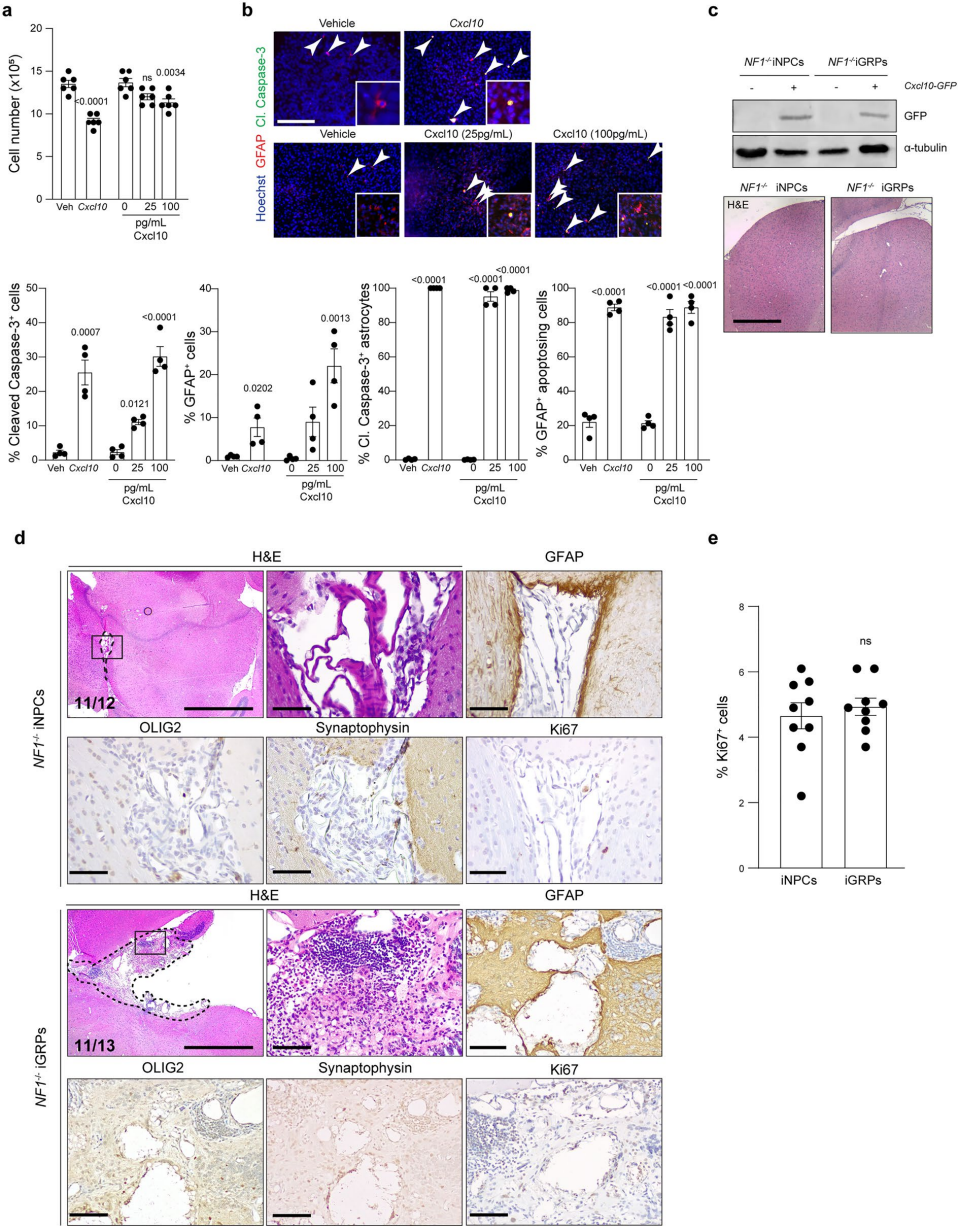
*Cxcl10* absence is both necessary and sufficient for LGG formation **a** *NF1-*null iNPC cell numbers decrease (direct cell count, top; 9–20% decrease) and the percent of cleaved caspase-3^+^ cells increases (bottom; 8.3–20.5-fold) following ectopic *Cxcl10* expression or incubation with 25 or 100 pg/mL of recombinant Cxcl10 protein. **b** Representative immunocytochemistry and quantification, demonstrating that ectopic *Cxcl10* expression or treatment with increasing concentrations of recombinant Cxcl10 peptide induce an increase in GFAP^+^ astrocytic differentiation (*Cxcl10*: 8.3-fold, 25 pg/mL Cxcl10: 8.2-fold and 100 pg/mL Cxcl10: 20.5-fold increase), while 95–100% of the differentiated GFAP^+^ cells are undergoing apoptosis (cleaved caspase-3^+^) and 83.3–88.8% of the total number of cells undergoing apoptosis (cleaved caspase-3^+^) are GFAP^+^. **c** Top panel: Ectopic expression of murine *Cxcl10* in 2041C > T^−/−^ *NF1*-null iNPCs and iGRPs (GFP expression, Western blot). α-tubulin was used as an internal protein loading control. Bottom panel: Ectopic murine *Cxcl10* expression inhibits LGG formation in *Rag1*^−/−^ mice at 1mpi (H&E images). Scale bar, 1 mm. **d** Representative images of H&E, GFAP, OLIG2, synaptophysin and Ki67 staining of 2041C > T^−/−^ *NF1*-null iNPC- and iGRP- derived LGGs in *Cxcl10*^*−/−*^ mice at 1mpi. The number of LGG-bearing mice is indicated within the H&E panels. Scale bars: left H&E panel, 1 mm; other panels, 100 µm. **e** Bar graphs denoting the percent of Ki67^+^ cells in the lesions. Data are represented as means ± SEM, **a** and **b** one-way ANOVA with Dunnett’s multiple comparisons test, **e** two-tailed student’s t-test. Individual *p* values are indicated within each graph. *ns* not significant

To determine whether Cxcl10 inhibits cell cycle progression or induces progenitor cell differentiation, *NF1-*null iNPCs were either engineered to ectopically express *Cxcl10* or incubated with increasing concentrations of recombinant murine Cxcl10 protein. Both treatments induced a decrease in cell number (ectopic *Cxcl10* expression, 20%; 25 pg/mL Cxcl10, 9%; 100 pg/mL Cxcl10, 13% decrease; Fig. 5a) and an increase in programmed cell death (cleaved caspase-3^+^ cells; ectopic *Cxcl10* expression, 9.4-fold; 25 pg/mL Cxcl10, 4.6-fold; 100 pg/mL Cxcl10, 20.5-fold increase; Fig. 5b). Additionally, both treatments increased GFAP^+^ astrocytic differentiation of the pluripotent iNPCs (ectopic *Cxcl10* expression, 8.3-fold; 25 pg/mL Cxcl10, 8.2-fold; 100 pg/mL Cxcl10, 20.5-fold increase; Fig. 5b). In this respect, 95–100% of the differentiated GFAP^+^ cells were cleaved caspase-3-positive, and 83.3–88.8% of the total number of cells undergoing apoptosis were astrocytes (Fig. 5b).Together, these results demonstrate that Cxcl10 induces astrocytic differentiation, as well as cell death, in vitro.

To demonstrate that stromal Cxcl10 is *sufficient* to inhibit LGG formation in vivo, *NF1-*null iNPCs and iGRPs engineered to ectopically express murine *Cxcl10* were injected into the brainstems of *Rag1*^−/−^ mice. In contrast to vector-infected controls, no LGGs formed in mice following the injection of *NF1-*null iNPCs and iGRPs expressing murine Cxcl10 (Fig. 5c). Conversely, to establish that Cxcl10 is *necessary* to inhibit LGG formation, we transplanted *NF1-*null iNPCs and iGRPs into the brainstems of *Cxcl10*^*−/−*^ mice. At 1 mpi, 92% and 85% of the injected mice developed iNPC- and iGRP-derived LGGs, respectively (Fig. 5d). These lesions were hypercellular, exophytic, and immunopositive for GFAP and OLIG2 expression, with 4.9% and 4.9% Ki67^+^ cells, respectively (Fig. 5e). Taken together, these data demonstrate that Cxcl10 is both necessary and sufficient to suppress LGG cell growth both in vitro and in vivo.

### Human pediatric LGG cell lines develop LGGs in ***Cxcl10***^***−/−***^ mice

**Fig. 6:**
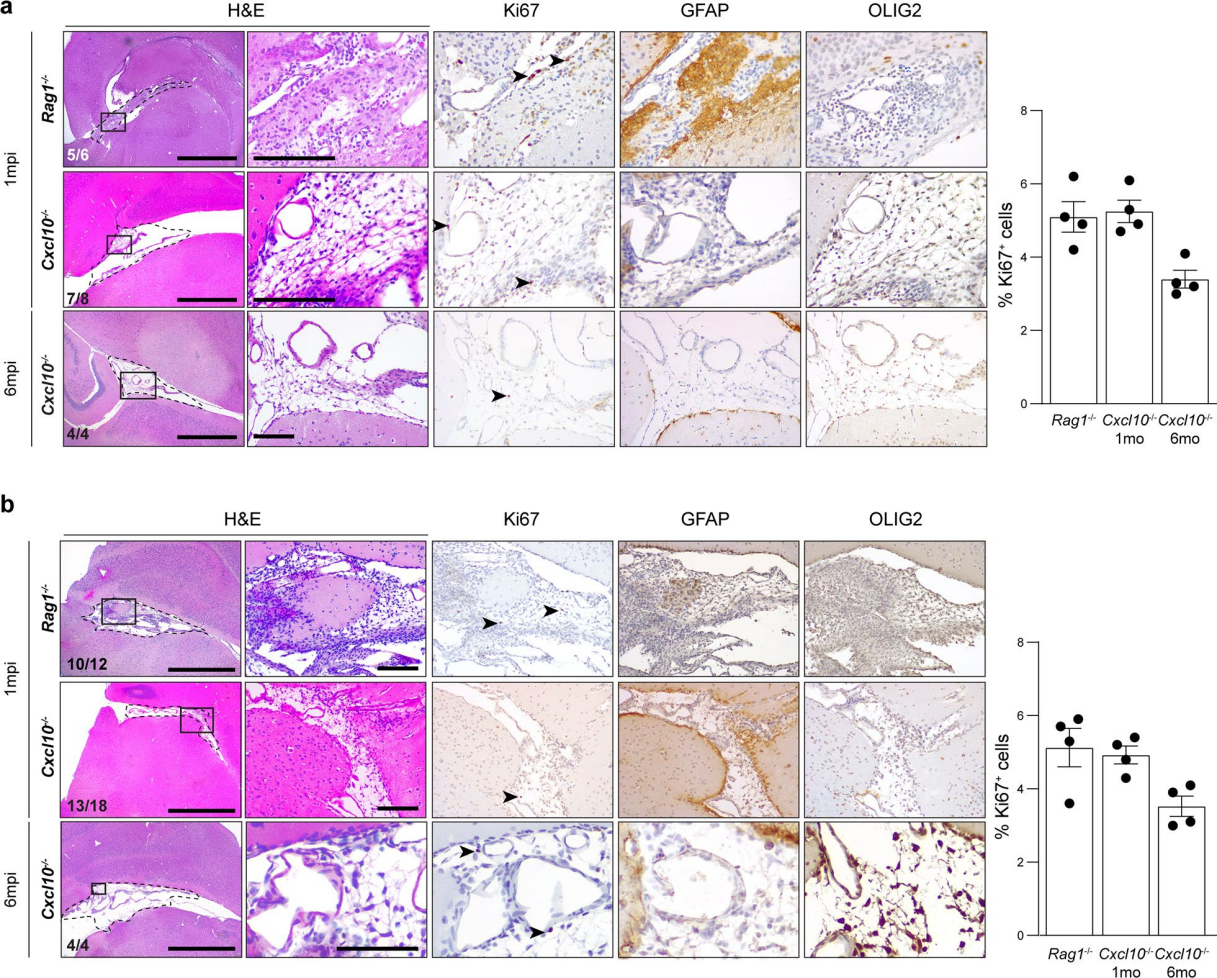
Pediatric LGG cells form lesions in *Rag1*^*−/−*^ and *Cxcl10*^*−/−*^ mice **a** and **b** Representative images of H&E, Ki67, GFAP and OLIG2 staining of pediatric LGG tumors in *Rag1*^*−/−*^ (top) and *Cxcl10*^*−/−*^ (bottom) mice at 1mpi and 6mpi using two pediatric LGG lines: JHH-NF-PA (**a**) and Res186 (**b**). The number of LGG-bearing mice is indicated within the H&E panels. Bar graphs denote the % Ki67^+^ cells in the lesions. Data are represented as means ± SEM. Scale bars: left H&E panel, 1 mm; other panels, 100 µm

To extend these findings to human PDX modeling, we leveraged two primary human PA cell lines from one patient with an NF1-PA (JHH-NF-PA; *NF1* loss) and another with a sporadic PA (Res186). While these lines grow briefly in larval zebrafish (6 days), they do not form tumors in athymic nude mice over the course of 12 months [65]. Similar to the experiments using hiPSC neuroglial lineage cells, 5 × 10^5^ PA cell lines were injected into brainstems of *Rag1*^*−/−*^ and *Cxcl10*^−/−^ mice. Whereas human pediatric PA cells did not form gliomas in wild type mice, both *Rag1*^*−/−*^ and *Cxcl10*^−/−^ mice developed LGGs at 1mpi and 6mpi. These LGGs were hypercellular with microcystic components, parenchymal with exophytic components, immunopositive for OLIG2, but mostly negative for GFAP, expression (Fig. 6). These lesions exhibited proliferative indices between 4.2 and 4.7% at 1mpi and 3.4–3.5% at 6 mpi. Together, these results establish *Cxcl10*^*−/−*^ mice as a tractable in vivo platform for human pediatric LGG modeling.

### hiPSC-LGG growth is reduced following MEK inhibition

**Fig. 7:**
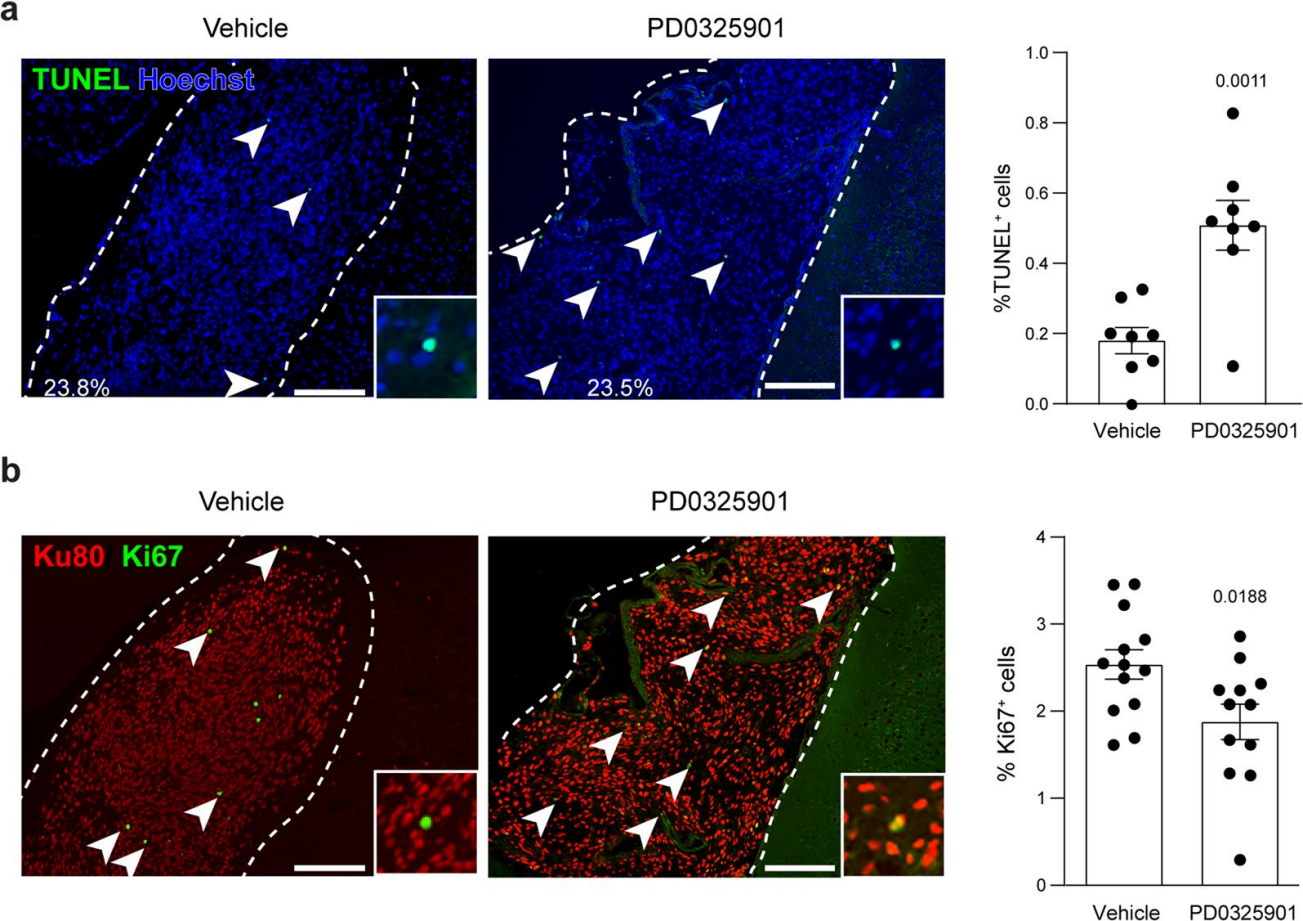
PD0325901 treatment induces apoptosis and decreases cell proliferation in iNPC-LGGs **a** and **b** TUNEL^+^ cells are increased (apoptotic cells; green; **a**), while Ki67^+^ cells are decreased (proliferating cells; green; **b**), in *NF1*^*−*/−^ iNPC-derived LGGs following PD0325901 treatment. Human tumor cells are Ku80^+^ (red; **b**). The % area occupied by the iNPC-LGGs is indicated within the images. Data are represented as means ± SEM, two-tailed student’s t-test; Individual *p* values are indicated within each graph. Scale bars, 50 µm

Finally, to provide a proof-of-principle demonstration that this humanized LGG platform could be used for preclinical drug evaluation, we assayed the ability of a MEK inhibitor (PD0325901) to block hiPSC-LGG growth beginning at 1 mpi in vivo. The treatment lasted a total of four weeks, a time frame we previously reported to inhibit *Nf1* mouse low-grade optic glioma growth [34]. While Ku80^+^ iNPC-LGGs were still detectable in mice following PD0325901 administration, there was increased tumor cell apoptosis (TUNEL^+^ cells), as well as reduced tumor cell proliferation (Ki67^+^ cells), relative to vehicle-treated LGGs (Fig. 7). In vitro PD0325901 treatment of *NF1* null iNPCs, iGRPs and iOPCs also resulted in decreased cell proliferation and increased apoptosis (Additional file 1: Fig. S7), thus establishing this experimental platform for preclinical therapeutic studies.

## Discussion

Building upon prior studies using hiPSC-derived 2D and 3D organoid cultures to study high-grade gliomas [1, 35, 39, 53] and medulloblastoma [63], we developed a humanized xenograft platform to model sporadic and NF1-associated pediatric LGGs and elucidate the pathogenesis, cellular origins, and signaling pathway dependencies. Beyond the value of this system to PDX pediatric LGG future preclinical experimentation, this study raises several important points germane to human brain tumor pathobiology.

First, leveraging hiPSC engineering for humanized tumor modeling represents an efficient system applicable to other low-grade nervous system tumor xenografts, which have been extremely challenging to establish in mice [28, 30, 49]. Relevant to pediatric LGGs, like NF1-PA and *BRAF*-driven PAs, the derivative tumor cells undergo oncogene-induced senescence [49] and display a senescence-associated secretory phenotype (SASP) in vitro unless provided with fibroblast conditioned medium and ROCK inhibition [65] or senolytic inhibitors [11]. The fragility of these PA tumor cell cultures likely reflects their profound stromal (tumor microenvironment) dependence, as well as their limited intrinsic self-renewal capacity [59], as demonstrated using genetically engineered mouse models and murine pediatric LGG explant systems. In these studies, *Nf1* optic glioma growth in mice requires T cell and microglia support through the elaboration of critical cytokines and growth factors, as *Nf1* optic glioma stem cells cannot form glioma-like lesions in mice lacking these stromal cells [22, 47]. Similarly, *KIAA1549:BRAF*-expressing cerebellar stem cells cannot form tumors in mice lacking the T cell and microglia *Ccr2* chemokine receptor [14]. Moreover, the SASP represents a cellular state characterized by the secretion of inflammatory cytokines and immune modulators, which may counter the pro-tumoral support provided by T cells and monocytes in the tumor microenvironment.

Second, the hiPSC-LGG explant system provides a tractable platform to define the putative cells of origin for histologically distinct tumors, as well as histologically similar tumors arising in different locations. This is particularly important with respect to the cellular ontogeny of brain cancers. In this regard, previous mouse modeling experiments have demonstrated that multiple cell types, including differentiated cells (astrocytes [6], neurons [17]), can give rise to high-grade gliomas. Using other murine modeling approaches, NPCs [37, 45, 60], or their derivative progenitor cells [2, 8], have been shown to be the putative cells of origin, while restricted progenitors of the NPC lineage give rise to oligodendrogliomas [48] and proneural glioblastomas [40]. Moreover, additional cellular constraints, including the specific neuroglial progenitor population [60] and the particular brain location (third ventricle versus lateral ventricle germinal zone [36]) are critical determinants that dictate glioma formation and latency. In this report, we leveraged specific human neuroglial lineage cells to determine that not all lineages faithfully recapitulate LGG histological features. Whereas astrocytes do not give rise to these tumors, iGRPs, iOPCs and iNPCs with *NF1* loss or *KIAA1549:BRAF* expression form LGGs within a month. It is intriguing to note that iGRPs give rise to more compact tumors with stronger GFAP than OLIG2 immunoreactivity, resembling optic pathway and brainstem gliomas, while the iOPC-derived lesions have looser stroma with microcystic changes and a higher density of OLIG2^+^ than GFAP^+^ cells, similar to many cerebellar human PAs. In contrast, Olig2^+^, NG2^neg^ glial progenitors form *Nf1* optic gliomas with delayed latency [60], which may reflect innate differences between neuroglial cell populations in humans and rodents and/or different potential cells of origin [38, 56].

Third, the finding that humanized pediatric LGGs develop in a mouse strain not lacking immune cells opens a new avenue for the in vivo study of low-grade PDXs. Whereas some orthotopically injected high-grade PDXs grow in wild type mice [25], the majority of PDX studies leverage immunocompromised (usually athymic and NOD/SCID) mice, due to their inherent inability to reject engrafted human cells. However, even in these immune-impaired animals, not all tumors are able to grow [25, 31], possibly due to the lack of a trophic environment provided by a complete immune system. Since T cells present in human PAs [13] and *Nf1* murine low-grade gliomas [22] establish a supportive microenvironment for low-grade glioma growth in vivo, it is important to use host systems with immune systems with limited immunologic impairment, such as *Cxcl10*^−/−^ mice. Additional studies are in progress to define immune function in *Cxcl10*-deficient mice.

The relevance of Cxcl10 production to xenograft establishment and tumorigenesis is further strengthened by prior reports demonstrating that elevated CXCL10 in bronchoalveolar lavage fluid is associated with acute lung transplant rejection [26] and that viral *CXCL10* gene therapy improves cervical cancer xenograft responses to radiotherapy [69]. In addition, increased Cxcl10 expression is inversely correlated with tumor growth and is associated with cardiac [24] allograft rejections in the PNS, such that enhanced systemic Cxcl10 expression is a prognostic biomarker of graft-versus-host disease [33]. Moreover, reduced Cxcl10 levels in HGG spheroids caused by long-term in vitro culture correlates with higher engraftment rate in immunocompetent rats [27]. Finally, CXCL10 is induced as part of the SASP [66], which limits primary human LGG cell growth in vitro through increased senescence [11, 49]. Understanding the mechanisms underlying CXCL10-mediated hiPSC- and patient-derived xenograft growth inhibition will be critical to the development of second-generation PDX models of pediatric LGGs.

## Conclusions

It should be appreciated that this xenograft model system has some inherent limitations. *Rag1*^*−/−*^ mice have impaired immune systems and the resulting LGGs lack some of the non-neoplastic cell types (e.g., T cells) important for murine LGG growth [3, 22, 46, 47]. Similarly, the impact of *Cxcl10* loss on immune system function is unknown. Ongoing studies are focused on co-introducing hiPSC-derived monocyte populations, as well as defining the impact of *Cxcl10* loss on non-neoplastic cell function. While additional work will be required to create preclinical models that fully recapitulate all of the elements of pediatric LGGs, we have generated an experimentally tractable in vivo platform for humanized orthotopic pediatric LGG modeling. Further refinement of this model has great potential to galvanize the study of a variety of different types of brain and nerve tumors, including previously unexplored low-grade subtypes, as well as advance our understanding of the molecular and cellular origins of these common brain tumors in children.

## Materials and methods

### Study approval

All experiments were performed under active and approved Animal Studies Committee protocols at Washington University.

### Animals

Mice were maintained on a 12 light/ dark cycle in a barrier facility, had ad libitum access to food and water, were exclusively used for the purposes of this study, and had not undergone any additional procedures. Mating cages housed one male and two females of the same genotype, and males were separated from dams at the time of delivery. Injections were performed on entire litters of 0–3-day-old mice of the following strains: C57BL/6J, *Rag1*^*−/−*^ (B6.129S7-*Rag1*^tm1Mom^/J; strain 002216, Jackson laboratories), *Cxcl10*^−/−^ (B6.129S4-*Cxcl10*^tm1Adl^/J; strain 006087, Jackson laboratories), CD4-deficient (B6.129S2-*Cd4*^*t*m1Mak^/J; strain 002663, Jackson laboratories), CD8-deficient (B6.129S2-*Cd8*^atm1Mak^/J; strain 002665, Jackson laboratories), CD4/CD8-deficient (intercrossed strains 002663 and 002665), *NOD*/*SCID* (NOD.Cg-Prkdc^scid^/J; strain 001,303, Jackson laboratories), *Ccl5*^−/−^ (B6.129P2-*Ccl5*^*tm1Hso*^/J; strain 005090; Jackson laboratories) and *Cx3cr1*^−/−^; *Ccr2*^−/−^ [47] mice. Injected pups were allowed to recover and were subsequently immediately returned to their maternal cage until weaning. Mice of both sexes were randomly assigned to all experimental groups without bias, and the investigators were blinded until final data analysis during all of the experiments.

### Human induced pluripotent stem cell culture and differentiation

NF1 patient homozygous and heterozygous germline *NF1* gene (Transcript ID NM_000267) mutations (c.2041C > T; c.6513T > A) were CRISPR/Cas9-engineered into a single commercially available male control human iPSC line (BJFF.6) by the Washington University Genome Engineering and iPSC Core Facility (GEiC). Homozygous mutations were confirmed by NGS sequencing [9], and two different clones were expanded for each of the *NF1*^*−/−*^ and control lines for all subsequent differentiation procedures. Human induced pluripotent stem cells (hiPSCs) were grown on Matrigel (Corning)-coated culture flasks, and were fed daily with mTeSR Plus medium (STEMCELL Technologies). hiPSCs were passaged as needed with ReLeSR medium (STEMCELL technologies) following the manufacturer’s instructions. For neural progenitor cell (iNPC) differentiation, hiPSCs were transferred to poly-l-ornithine (Sigma-Aldrich)/Laminin (Fisher)-coated tissue culture flasks and incubated for 3 days in NPC basic media [50% DMEM/F12, 50% Neurobasal medium, 1 × N-2 supplement, 1 × B-27 supplement, 2 mM GlutaMax (all Thermo Fisher Scientific)] supplemented with 10 ng/mL human LIF, 4 μM CHIR99021, 3 μM SB431542, 2 μM Dorsomorphin and 0.1 μM Compound E (all STEMCELL Technologies). Subsequently, cells were incubated for 5 days in NPC basic medium supplemented with 10 ng/mL human LIF, 4 μM CHIR99021, 3 μM SB431542, and 0.1 μM Compound E. Finally, iNPCs were incubated and maintained in NPC basic medium supplemented with 10 ng/mL human LIF, 3 μM CHIR99021 and 2 μM SB431542. The medium was refreshed daily, and iNPCs passaged as needed with Accutase (STEMCELL Technologies) following the manufacturer’s instructions. For astrocyte differentiation, iNPCs were transferred onto Primaria-coated plates and maintained in Astrocyte growth medium (ThermoFisher Scientific). Astrocytes were fed 3 times a week and passaged as needed with 0.05% Trypsin (Fisher) following the manufacturer’s instructions. For glial restricted progenitor (iGRP) differentiation, iNPCs were dissociated with Accutase (STEMCELL Technologies) following the manufacturer’s instructions, and floating cells transferred to low-attachment culture flasks to allow for gliosphere formation. iGRPs were incubated for 2 weeks in the following medium: Basal GRP medium [DMEM/F12 supplemented with Sodium bicarbonate (Sigma-Aldrich), 1 × B-27 Supplement (Thermo Fisher Scientific), 1 × N-2 Supplement (Thermo Fisher Scientific), 1% penicillin–streptomycin (Thermo Fisher), 1% L-glutamine (Thermo Fisher Scientific), and 1% nonessential amino acids (Thermo Fisher Scientific)] supplemented with 10 ng/mL NT-3 (Peprotech), 10 μM forskolin (Tocris), 60 ng/mL 3,3′,5-triiodo-l-thyronine (T3; Sigma-Aldrich), 20 μg/mL ascorbic acid (Sigma-Aldrich) and 25 μg/mL insulin (Sigma-Aldrich). The supernatant was refreshed 3 times a week by gentle aspiration. For subsequent glial differentiation, gliospheres were incubated for 3 weeks in basal GRP medium supplemented with 20 ng/mL PDGF-AA (Peprotech), 10 ng/mL IGF-1 (Fisher), 10 ng/mL NT-3 (Peprotech), 10 μM forskolin (Tocris), 60 ng/mL T3 (Sigma-Aldrich), and 10 μg/mL insulin (Sigma-Aldrich). The supernatant was refreshed 3 times a week by gentle aspiration. For oligodendrocyte progenitor cell (OPC) differentiation, embryoid bodies (EBs) were generated directly from iPSCs by seeding 60,000 iPSCs at the bottoms of ultra-low cell attachment U-bottom 96 well plates, and incubating them for 5 days in NIM (DMEM/F12, 1% NEAA, 1 × N-2 supplement). Subsequently, the EBs were transferred onto poly-l-ornithine/Laminin-coated 6-well plates and incubated for 11 days in NIM supplemented with 20 ng/mL bFGF (Peprotech) and 2 µg/mL heparin (STEMCELL technologies), 3 days in NIM supplemented with 100 nM retinoic acid (RA; Sigma-Aldrich), 7 days in NIM supplemented with 100 nM RA, 1 µM Purmorphamine (Pur; STEMCELL Technologies) and 1 × B-27, and then 11 days incubation in NIM supplemented with 10 ng/mL bFGF, 1 µM Pur and 1 × B-27. For OPC specification and maturation, the spheres were then transferred to low-attachment culture flasks and incubated for 120 days in glial induction medium [DMEM/F12, 1 × N1 (Sigma-Aldrich), 1 × B27, 60 ng/mL T3, 100 ng/mL Biotin (Sigma-Aldrich) and 1 µM cAMP (Peprotech)] supplemented with 10 ng/mL PDGF-AA, 10 ng/mL IGF-1 and 10 ng/mL NT3.

### Intracranial injections

Postnatal day 0–4 animals were anaesthetized and intracranially injected in accordance with active Animal Studies Committee protocols at Washington University. 1 × 10^4^, 5 × 10^4^, 1 × 10^5^ or 5 × 10^5^ cells resuspended in 2 µL ice-cold PBS were injected 0.7 mm to the right of the midline into either the midbrain (0.5 mm posterior to Lambda; 2 mm deep), or the cerebellum (2 mm posterior to Lambda; 1 mm deep) of neonatal mice using a Hamilton syringe. Animals were aged to 1, 3 or 6 months post-injection prior to tissue harvesting and analysis.

### PD0325901 treatments

Twenty 4-week-old *Rag1*^−/−^ mice of both sexes harboring *NF1-*null iNPC-LGGs 1 month post-injection were intraperitoneally injected either with 10% DMSO in saline or with 5 mg/kg/day PD0325901 (Sigma-Aldrich), 6 days a week, for a total of four weeks [34]. Treated mice were collected for histopathologic analysis. For in vitro experiments, iNPCs, iGRPs and iOPCs were treated with 10 nM PD0325901 for 24 h prior to immunocytochemical analysis.

### Magnetic resonance imaging (MRI)

Injected mice were transcardially perfused with Ringer’s solution and 4% paraformaldehyde (PFA). The entire brain was removed from the animals and post-fixed in 4% PFA overnight prior to rehydration in phosphate buffered saline (PBS) for a minimum of 7 days. Rehydrated brain samples underwent MRI. Each mouse brain was then packed into a 2 mL plastic vial, supported with fiberglass and immersed in Fluorinert (FC-3283; 3 M Company, St. Paul, MN). MRI experiments were performed using a 4.7-T small-animal MR scanner built around an Agilent/Varian (Santa Clara, CA) DirectDriveTM console and an Oxford Instruments (Oxford, United Kingdom) horizontal-bore superconducting magnet. The plastic vial containing the mouse brain was loaded into a laboratory-built solenoid RF coil (1-cm diameter; 2-cm length). MR images were collected with a 3D gradient echo (GE3D) sequence: TR 5.0 ms, TE 2.2 ms, flip angle 30°, matrix size 128 × 128 × 128, FOV 16 × 16 × 16 mm^3^, 0.19 mm isotropic resolution, 4 averages, and 5.5 min data acquisition time. The images were loaded into MatLab (MathWorks®, Natick, MA) and converted into NIfTI (.nii) format for tumor inspection and segmentation with ITK-SNAP (www.itksnap.org).

### Tissue fixation and immunohistochemistry

Injected mice were transcardially perfused at 1, 3 or 6 months post-injection, initially with Ringer’s solution, and then followed by ice-cold 4% paraformaldehyde. Whole mouse brains were harvested, post-fixed in 4% PFA and dehydrated in 70% ethanol. Dehydrated samples were then paraffin-embedded and serially sectioned (5 µm). Hematoxylin and eosin (H&E), as well as antibody immunohistochemical staining, were performed using the primary antibodies, Vectastain ABC kit (Vector Laboratories) and appropriate biotinylated secondary antibodies provided in Additional file 1: Table S1. Immunostaining was performed using the primary antibodies listed in Additional file 1: Table S1 with appropriate Alexa Fluor-conjugated secondary antibodies. Bis-benzamide (Hoechst) was used as a nuclear counterstain.

### Immunocytochemistry

Immunocytochemistry was performed on hiPSCs, iNPCs, iGRPs, iOPCs and astrocytes using the primary antibodies described in Additional file 1: Table S1. Briefly, adherent cells were washed, fixed with 4% paraformaldehyde, permeabilized with 0.5% Triton X-100 in PBS, before blocking in 10% goat serum and incubation overnight at 4 °C in primary antibodies diluted in 2% goat serum at the manufacturer’s suggested concentrations. Appropriate secondary Alexa-Fluor-conjugated secondary antibodies diluted to 1:200 were employed and bis-benzamide (Hoechst) was used as a nuclear counterstain. Cells were mounted with Immu-mount (Fisher) and imaged on a Leica DMi1 fluorescent microscope using the Leica LAS X software, as per manufacturer’s instructions.

### BrdU proliferation, RAS activity, cAMP and Cxcl10 ELISA

RAS activity (ThermoFisher), cAMP (Fisher) detection, BrdU proliferation assays (Roche) and direct cell counting were performed according to the manufacturer’s instructions. Cxcl10 (Ray Biotech) ELISA assays were performed according to the manufacturer’s instructions. Each assay was performed using a minimum of three independently generated biological replicates.

### Lentivirus production, cell infection and CXCL10 peptide treatment

*Cxcl10* cDNA (Sino Biological) and lentiviral packaging vectors, or *KIAA1549:BRAF* adenoviral DNA, were transfected in HEK293T cells using Fugene HD (Promega) following the manufacturer’s instructions. Viral supernatants were collected 48 h post-transfection, filtered and used to directly infect iNPCs and iGRPs for 24 h. Cxcl10-GFP expression was confirmed by Western blotting as previously described (Anastasaki et al., 2015). *KIAA1549:BRAF*-infected cells were puromycin-selected for 2 weeks prior to further expansion. Infected cells were used for intracranial injections in *Rag1*^*−/−*^ neonatal mice. For cell proliferation, cell death and differentiation analyses, iNPCs were treated with 25 or 100 pg/mL of murine recombinant Cxcl10 (PeproTech) for 24 h. Each experiment was performed a minimum of three times on independently generated samples.

### RNA extraction, quantitative real-time PCR and RNA sequencing and analysis

RNA was extracted using a QIAGEN RNeasy mini-kit from snap-frozen brainstem tissues of 1-month-old adult mice following the manufacturer’s instructions (QIAGEN). For quantitative real-time PCR (qPCR) studies, total RNA was reverse-transcribed using the High-Capacity cDNA Reverse Transcription Kit protocol following the manufacturer’s instructions (Thermo Fisher Scientific). qPCR was performed on a Bio-Rad CFX thermocycler using pre-designed TaqMan Gene Expression Assays (*Cxcl10*, *Chil3*, *CD59a*, *Gfap*; Additional file 1: Table S2) and a commercially available Taqman mastermix (Thermo Fisher Scientific) following the manufacturer’s instructions. Relative transcript expression was calculated using the ΔΔCT analysis method and normalized to *Gapdh* as an internal control following the manufacturer’s instructions (ThermoFisher). For RNA sequencing, total RNA from three C57BL/6J and three *Rag1*^*−/−*^ brainstem samples was submitted to Washington University Genome Technology Access Center (GTAC). Samples were prepared according to the library kit manufacturer’s protocol, indexed, pooled, and sequenced on an Illumina HiSeq. Basecalls and demultiplexing were performed with Illumina bcl2fastq software and a custom python demultiplexing program with a maximum of one mismatch in the indexing read. The analysis was generated using Partek Flow software, version 8.0. RNA sequencing reads were aligned to the mm10—RefSeq Transcripts 83 assembly with STAR version 2.5.3a. Gene counts and isoform expression were derived from the annotation model output. Sequencing performance was assessed for the total number of aligned reads, total number of uniquely aligned reads, and features detected. Normalization size factors were calculated for all gene counts by CPM to adjust for differences in library size. Gene specific analysis was then performed using the lognormal with shrinkage model (limma-trend method) to analyze for differences between *Rag1*^−/−^ and control C57 mice. The results were filtered for only those genes with *p* values ≤ 0.05 and log fold-changes ≥  ± 2. Principal component analysis was conducted in Partek Flow using normalized gene counts. RNA sequencing data have been deposited in the GEO portal (GSE174624).

### T cell isolation and culture

4–6-week-old C57BL/6J and *Rag1*^−/−^ mouse spleens were homogenized into single cell suspensions by digestion in PBS containing 0.1% BSA and 0.6% sodium citrate. The homogenates were subsequently washed and incubated with 120 Kunitz units of DNase I for 15 min following red blood cell lysis (eBioscience). Cells were then filtered through a 30 µM cell strainer to obtain a single cell suspension. CD4^+^ and CD8^+^ T cells were isolated using CD8a (Miltenyi Biotech) or CD4 (Miltenyi Biotech) T cell isolation kits, respectively. T cells were maintained at 2.5 × 10^6^ cells ml^−1^ in RPMI-1640 medium supplemented with 10% FBS and 1% penicillin/ streptomycin. T cells were activated by 1.25 µg ml^−1^ anti-mouse CD3 (Fisher Scientific) and 2 µg ml^−1^ anti-mouse CD28 (Fisher Scientific) antibody treatment for 48 h. T cell conditioned media (TCM) was collected both from non-activated and activated T cells following 22 µM filtration for subsequent chemokine assay, ELISA and co-culture experiments.

### Astrocyte isolation and culture

4- to 6-week-old C57BL/6 J and *Rag1*^−/−^ mice were transcardially perfused with DPBS and whole brains collected following the removal of the cerebellum and olfactory bulbs. A single cell suspension was generated using the Miltenyi Biotech adult brain dissociation kit following the manufacturer’s instructions. The resulting cells were seeded in Poly-l-Lysine-coated (Millipore Sigma) T75 flasks and incubated in Minimal Essential Medium supplemented with 1 mM l-glutamine, 1 mM sodium pyruvate, 0.6% D-(+)-glucose, 100 μg ml^−1^ penicillin/ streptomycin and 10% FBS. A complete media change was performed after 24 h and every third day after that for 12–14 days, in order to obtain mixed glial cultures composed of microglia on an astrocyte monolayer. To release the microglia, flasks were mechanically shaken at 200 rpm for 5 h at 37 °C, and the microglia-containing supernatant discarded. Astrocyte-enriched cultures containing > 95% GFAP-positive astrocyte monolayers were passaged with 0.1% trypsin (Invitrogen) for subsequent experiments. Conditioned media from naïve or TCM-treated astrocytes was collected following 22 µM filtration.

### Quantification and statistical analysis

All statistical tests were performed using GraphPad Prism 5 software. 2-tailed Student’s t-tests, or one-way analysis of variance (ANOVA) with Bonferroni post-test correction using GraphPad Prism 5 software. Statistical significance was set at *p* < 0.05, and individual *p* values are indicated within each graphical figure. A minimum of 3 independently generated biological replicates was employed for each of the analyses. Numbers (n) are noted for each individual analysis.

## Supplementary Information

**Additional file 1**.

## Data Availability

All data generated or analyzed during this study are included in this published article and its supplementary information files. RNA sequencing data have been deposited in the GEO portal (GSE174624).
