# Supplementary material for: Human induced pluripotent stem cell engineering establishes a humanized mouse platform for pediatric low-grade glioma modeling
Source: Acta Neuropathol Commun. 2022 Aug 19;10:120. doi: 10.1186/s40478-022-01428-2 (PMC9392324; doi:10.1186/s40478-022-01428-2)
Supplement: Supplementary file 1 — Additional file 1. [file 40478_2022_1428_MOESM1_ESM.pdf]

Additional File 1 - Supplementary Information

Human induced pluripotent stem cell engineering establishes a humanized mouse platform for pediatric low-grade glioma modeling

Corina Anastasaki[1], Jit Chatterjee[1], Olivia Cobb[1], Shilpa Sanapala[1], Suzanne M. Scheaffer[1], Amanda De Andrade Costa[1], Anna F. Wilson[1], Chloe M. Kernan[1], Ameera H. Zafar[1], Xia Ge[2], Joel R. Garbow[2], Fausto J. Rodriguez[3], David H. Gutmann[1]

Departments of [1]Neurology and [2]Radiology, Washington University, St. Louis, MO 63110 USA; [3]Department of Pathology, David Geffen School of Medicine at UCLA, Los Angeles, CA 90095 USA

Tables

Additional File 1: Table S1. Antibodies used.

Additional File 1: Table S2. Primers used.

Figures

Additional File 1: Fig. S1 Characterization of hiPSCs and iNPCs

Additional File 1: Fig. S2 Analysis of hiPSC-NPC-injected *Rag1*$^{-/-}$ mice

Additional File 1: Fig. S3 *In vitro* characterization of iGRPs, iOPCs, and astrocytes

Additional File 1: Fig. S4 Immunostaining of *KIAA1549:BRAF*-expressing iGRP and iOPC LGGs

Additional File 1: Fig. S5 Analysis of iNPC-injected mice and RNA expression in different genetically engineered mouse strains

Additional File 1: Fig. S6 Immunostaining of naïve mouse brainstems

Additional File 1: Fig. S7 In vitro treatment of iNPCs, iGRPs and iOPCs with PD0325901

Additional File 1: Fig. S8 Uncropped western blot images

Additional File 1: Table S1 Antibodies used

Antibody	Manufacturer	Catalog Number
Anti-ABCG1 antibody	GeneTex	GTX30598
Anti-ALDH1L1 antibody	Abcam	ab87117
Anti-Alpha-1 Fetoprotein (AFP) antibody [EPR9309]	Abcam	ab169552
Anti-cleaved caspase-3 (Asp175) antibody	Cell Signaling Technologies	9661S
Anti-CD133 antibody	Abcam	ab19898
Anti-CD28 antibody	Fisher Scientific	16-0281-82
Anti-CDKN2A/p16INK4a antibody [2D9A12]	Abcam	ab54210
Anti-EAAT1 antibody	Abcam	ab416
Anti-EAAT2 antibody	Abcam	ab203130
Anti-GFAP/Glial Fibrillary Acid Protein antibody (2.2B10)	Fisher	13-0300
Anti-Ki67, Clone B56	BD Biosciences	BDB556003
Anti-Ku80 (C48E7) antibody	Cell Signaling	2180S
Anti-MBP antibody	Abcam	ab62631
Anti-Nestin antibody (ICC)	Abcam	ab92391
Anti-Nestin antibody (IHC)	Abcam	ab18102
Anti-NG2 antibody	Abcam	ab129051
Anti-O4 antibody, clone 81	Fisher Scientific	MAB345MI
Anti-OLIG2 antibody	GenTex	GTX132732
Anti-p44/42 MAPK (Erk1/2) antibody	Cell Signaling Technologies	9102S
Anti-Phospho-p44/42 MAPK (Erk1/2) (Thr202/Thr204) antibody	Cell Signaling Technologies	9101S
Anti-PDPN antibody	Abcam	ab236529
Anti-S100β antibody	Abcam	ab52642
Anti-Smooth Muscle Actin (SMA) antibody	Abcam	ab265588
Anti-SOX2 antibody	Abcam	ab92494

Anti-SOX10 antibody [SOX10/991]	Abcam	ab212843
Anti-Synaptophysin antibody	Abcam	ab32127
Anti-α Tubulin antibody	Cell Signaling Technologies	3873S
Anti-β III Tubulin antibody [2G10]	Abcam	ab78078
Alexa Fluor 488 goat anti-mouse antibody	Fisher Scientific	A11029
Alexa Fluor 568 goat anti-rabbit antibody	Fisher Scientific	A11011
Alexa Fluor 647 goat anti-mouse antibody	Abcam	ab150115
Biotinylated anti mouse secondary antibody	Fisher Scientific	BA9200
Biotinylated anti rabbit secondary antibody	Vector Laboratories	BA-1000
Senescence β-Galactosidase staining kit	Cell Signaling Technologies	9860S
StemLight™ Pluripotency antibody kit [OCT-4A (C30A3), SOX2 (D6D9), NANOG (D73G4) XP, SSEA (MC813), TRA-1-60, TRA-1-81)]	Cell Signaling Technologies	9656S

Additional File 1: Table S2 Primers used

**Primer**	**Manufacturer**	**Catalog Number**
Mouse *CD59a* - TaqMan® Gene Expression Assay FAM-MGB	ThermoFisher Scientific	Mm00483149_m1
Mouse *Chil3* - TaqMan® Gene Expression Assay FAM-MGB	ThermoFisher Scientific	Mm00657889_mH
Mouse *Cxcl10* - TaqMan® Gene Expression Assay FAM-MGB	ThermoFisher Scientific	Mm00445235_m1
Mouse *Gapdh* - TaqMan® Gene Expression Assay FAM-MGB	ThermoFisher Scientific	Mm99999915_g1

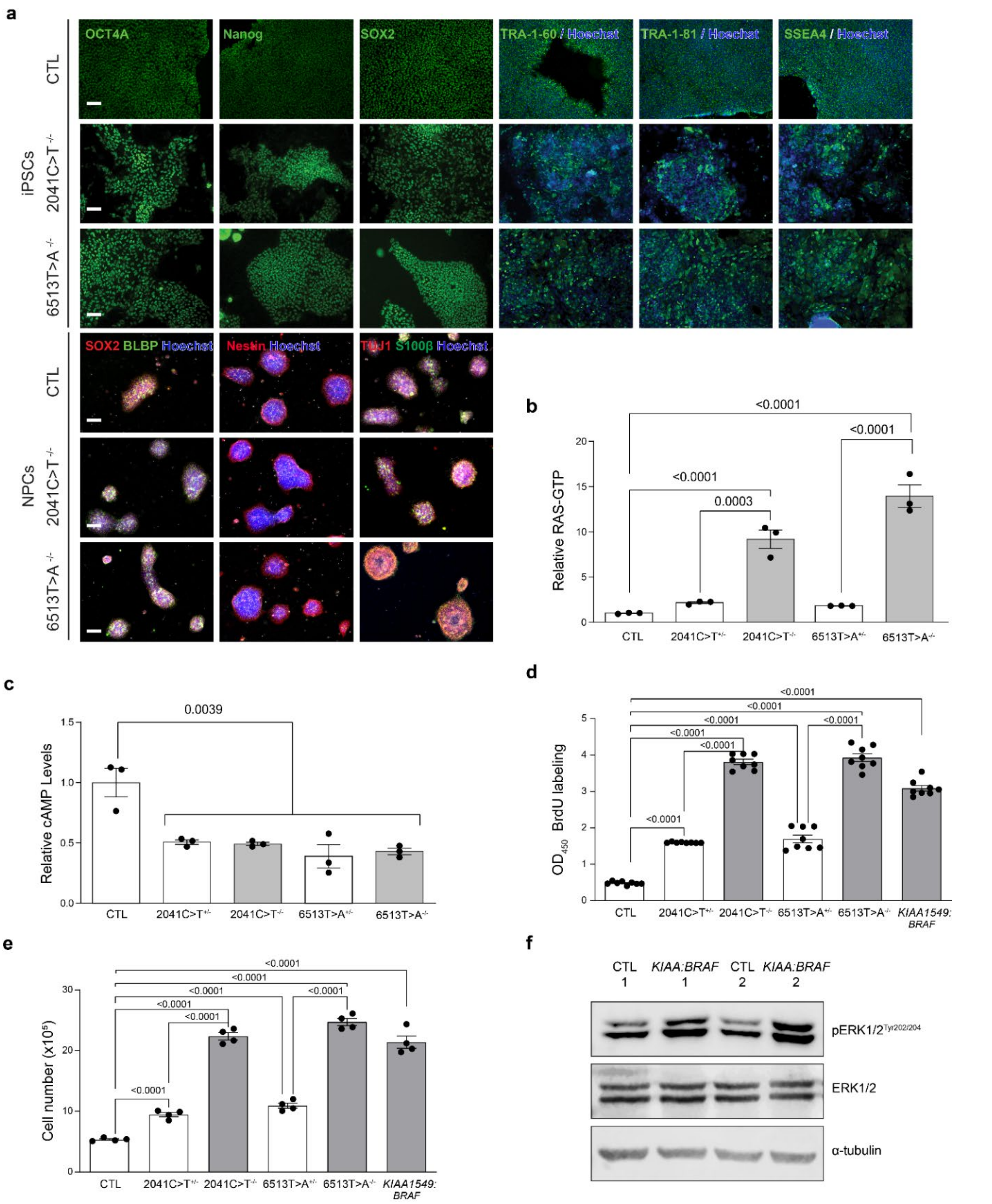

Additional File 1: Fig. S1 Characterization of hiPSCs and iNPCs (**a**) Top panel: *NF1*-null (2041C>T$^{-/-}$, 6513T>A$^{-/-}$) and control hiPSCs are immunopositive for OCT4A, Nanog, SOX2, TRA-1-60, TRA-1-81, and SSEA4 (pluripotency markers) expression. Bottom panel: 2041C>T$^{-/-}$, 6513T>A$^{-/-}$ and control iNPCs

express SOX2, BLBP and Nestin, and can be differentiated into TUJ1^{+} neurons and S100β^{+} glial cells. Scale bars, 25μm. (**b**) RAS activity is increased by 2.2- and 1.8-fold in heterozygous *NF1*-mutant iNPCs (2041C>T$^{+/-}$ and 6513T>A$^{+/-}$), respectively, and by 9.2- and 13.9-fold in *NF1*-null iNPCs (2041C>T$^{-/-}$ and 6513T>A$^{-/-}$), respectively, compared to controls. n=3. (**c**) cAMP levels are equivalently reduced in heterozygous *NF1*-mutant (2041C>T$^{+/-}$: 49%; 6513T>A$^{+/-}$: 61%) and *NF1*-null (2041C>T$^{-/-}$: 51%; 6513T>A$^{-/-}$: 57%) iNPCs relative to controls. n=3. (**d-e**) BrdU incorporation (proliferation; **d**; n=8) is increased 3.3- and 3.5-fold in heterozygous *NF1*-mutant iNPCs (2041C>T$^{+/-}$ and 6513T>A$^{+/-}$) iNPCs, 7.9- and 8.2-fold in *NF1*-null iNPCs (2041C>T$^{-/-}$ and 6513T>A$^{-/-}$) and 6.5-fold in *KIAA1549:BRAF* iNPCs, respectively, compared to controls. Direct cell counting (**e**; n=4) reveals a 1.8- and 2.0-fold increase in heterozygous *NF1*-mutant iNPCs (2041C>T$^{+/-}$ and 6513T>A$^{+/-}$), a 4.2- and 4.6-fold increase in *NF1*-null iNPCs (2041C>T$^{-/-}$ and 6513T>A$^{-/-}$), and 4-fold in *KIAA1549:BRAF* iNPCs relative to controls. (**f**) Western immunoblots demonstrate increased phospho-ERK1/2 normalized to total ERK1/2 in *KIAA1549:BRAF*-iNPCs and isogenic controls (CTLs). Two independently generated clones for each of the iNPC lines are included. α-tubulin is used as a protein loading control. All data are represented as means ±SEM; one-way ANOVA with Bonferroni post-test correction. Individual p values are indicated within each graph.

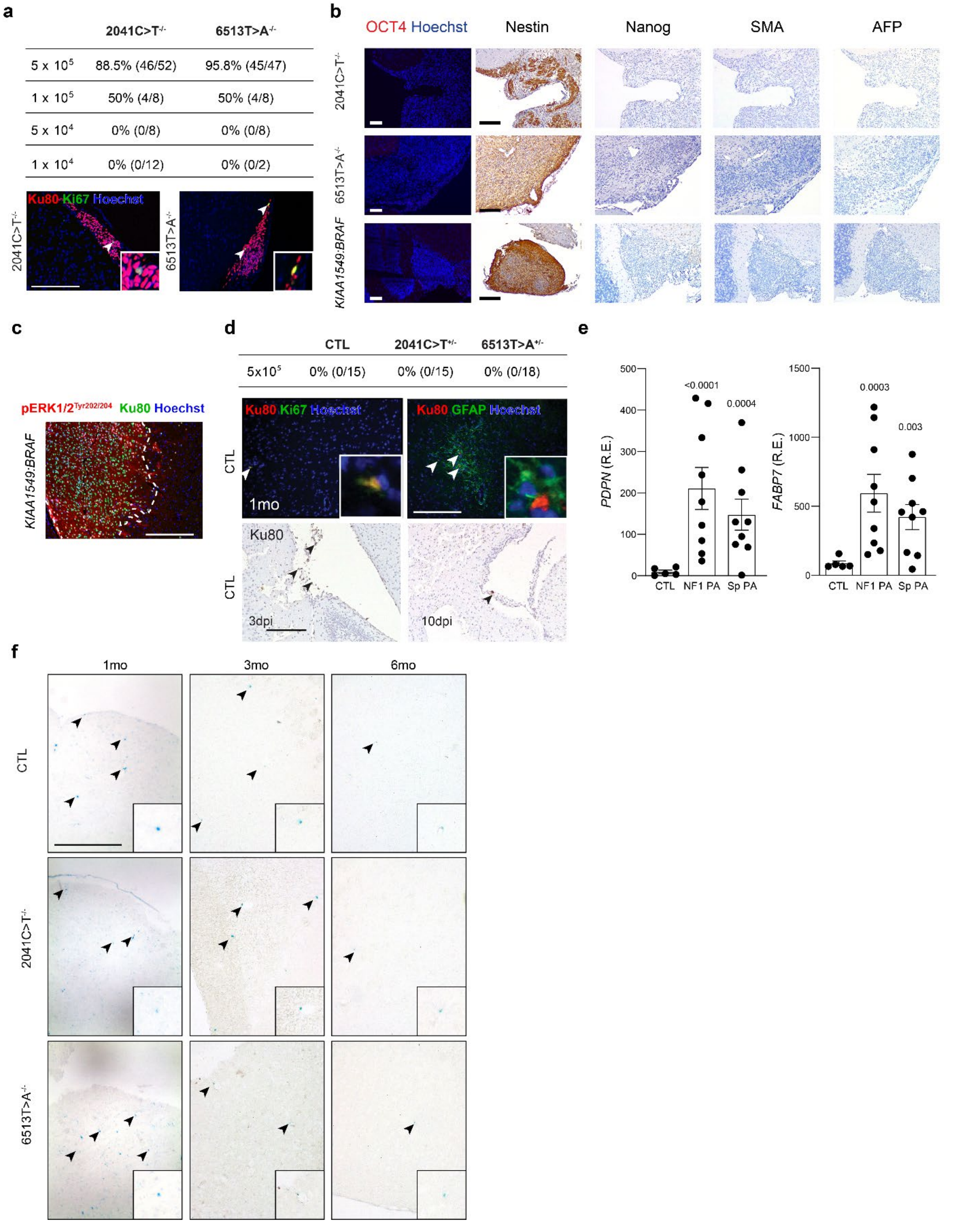

Additional File 1: Fig. S2 Analysis of iNPC-injected *Rag1*$^{-/-}$ mice (**a**) Summary of the percentages of *Rag1*$^{-/-}$ mice harboring LGGs one month after injection of 5x10^5, 1x10^5, 5x10^4 and 1x10^4 2041C>T$^{-/-}$ or

6513T>A$^{-/-}$ *NF1*-null iNPCs. The total number of mice injected is shown in the parentheses. Lower panel: representative images of Ku80^{+} and Ki67^{+} cells in *Rag1*$^{-/-}$ mouse brainstems one month after injection of 1x10^{5} 2041C>T$^{-/-}$ and 6513T>A$^{-/-}$ *NF1*-null iNPCs. White arrowheads indicate Ku80^{+} (human) cells. (b) iNPC LGGs are negative for non-specific pluripotency markers OCT4 and Nanog, negative for endoderm and mesoderm markers SMA and AFP, and immunopositive for neural progenitor pluripotency marker Nestin. (**c**) Increased ERK1/2 phosphorylation (activation; pERK1/2, red) is observed in *KIAA1549:BRAF*-iNPC LGG tumor cells (Ku80^{+} cells, green) relative to the surrounding normal brain tissue (Ku80-negative). Dashed white lines indicate the lesion area. (**d**) *PDPN* and *FABP7* are differentially expressed in NF1-associated and sporadic (Sp-PAs) pilocytic astrocytomas (PAs) relative to non-neoplastic brain tissue (CTL). R.E., relative expression. (**e**) *Rag1*$^{-/-}$ mice do not develop LGGs 1 month post-injection (m.p.i.) of 5x10^{5} CTL, 2041C>T$^{+/-}$ and 6513T>A$^{+/-}$ (heterozygous *NF1*-mutant) iNPCs. The lower panel shows representative images of Ku80^{+}, Ki67^{+} and GFAP^{neg} CTL iNPCs at the identified injection site. White arrowheads indicate Ku80^{+} iNPCs. (**f**) There is no increase in beta-galactosidase^{+} (senescent) cells in *Rag1*$^{-/-}$ brainstems injected with control (top panels; CTL; 0.3%) or 2041C>T$^{-/-}$ and 6513T>A$^{-/-}$ (*NF1*-null; lower panels; 0.3-0.4%) iNPCs at 1, 3 or 6mpi. Scale bars, **c-e** 50μm, **b**, **f** 100μm.

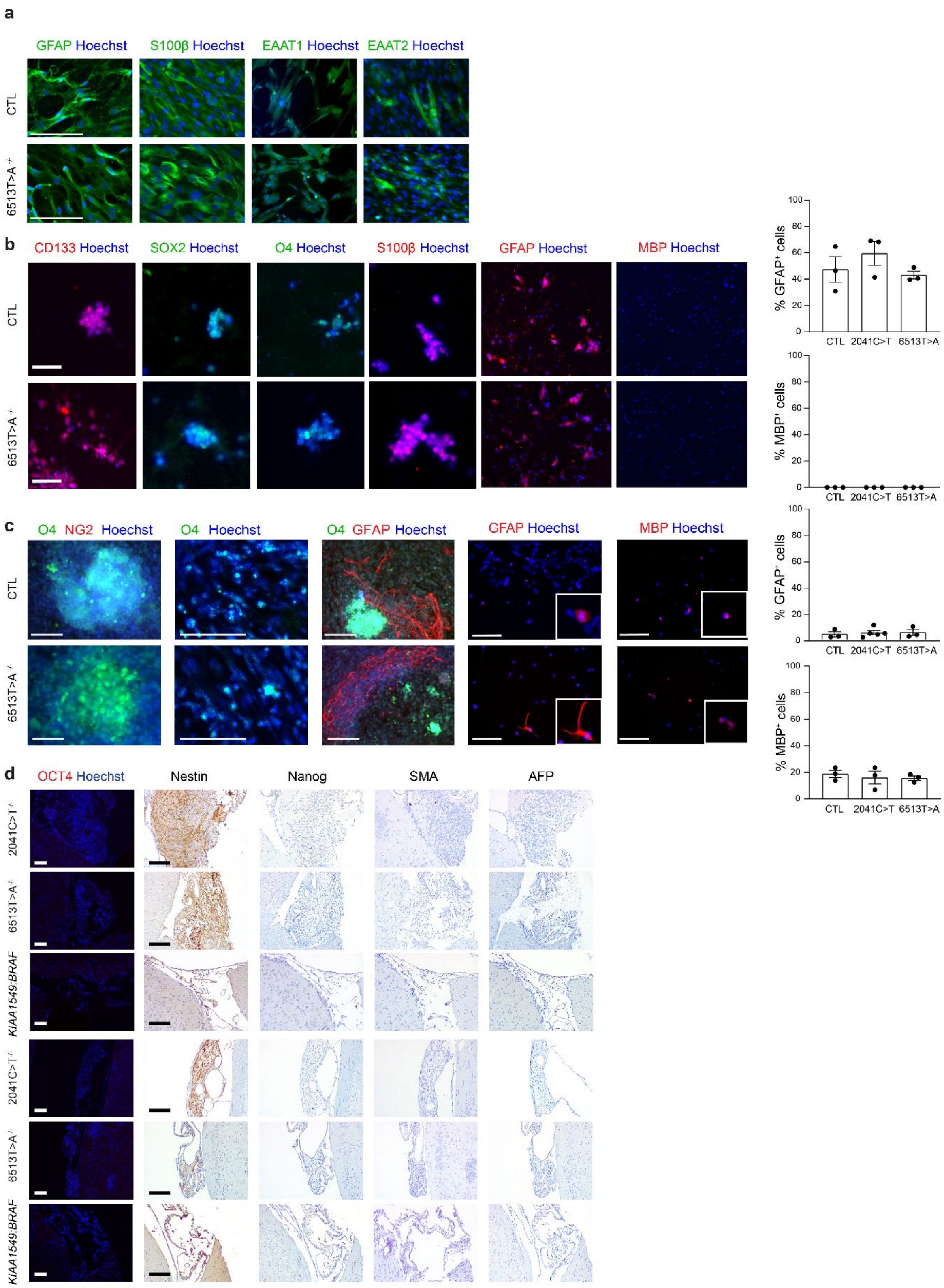

Additional File 1: Fig. S3 *In vitro* characterization of iGRPs, iOPCs, and astrocytes (**a**) CTL and 6513T>A$^{-/-}$ astrocytes are GFAP^{+}, S100β^{+}, EAAT1^{+} and EAAT2^{+} cells. (**b**) (left) CTL and 6513T>A$^{-/-}$

iGRPs are $CD133^+$, $SOX2^+$, $ABCG1^+$, $O4^+$, $GFAP^+$ and $S100\beta^+$, but MBP^{neg} cells. (right) Quantification of the percentage of GFAP- and MBP-immunopositive iGRPs. (**c**) CTL and $6513T>A^{-/-}$ iOPCs are $O4^+$, MBP^+, $GFAP^+$ and $NG2^{neg}$ cells. (right) Quantification of the percentage of GFAP- and MBP-immunopositive iOPCs. Data are shown as means ± SEM. (**d**) iGRP- and iOPC-LGGs are immunonegative for non-specific pluripotency markers (OCT4 and Nanog) and endoderm and mesoderm markers (SMA and AFP), but immunopositive for the neural progenitor pluripotency marker Nestin. All scale bars, 100μm.

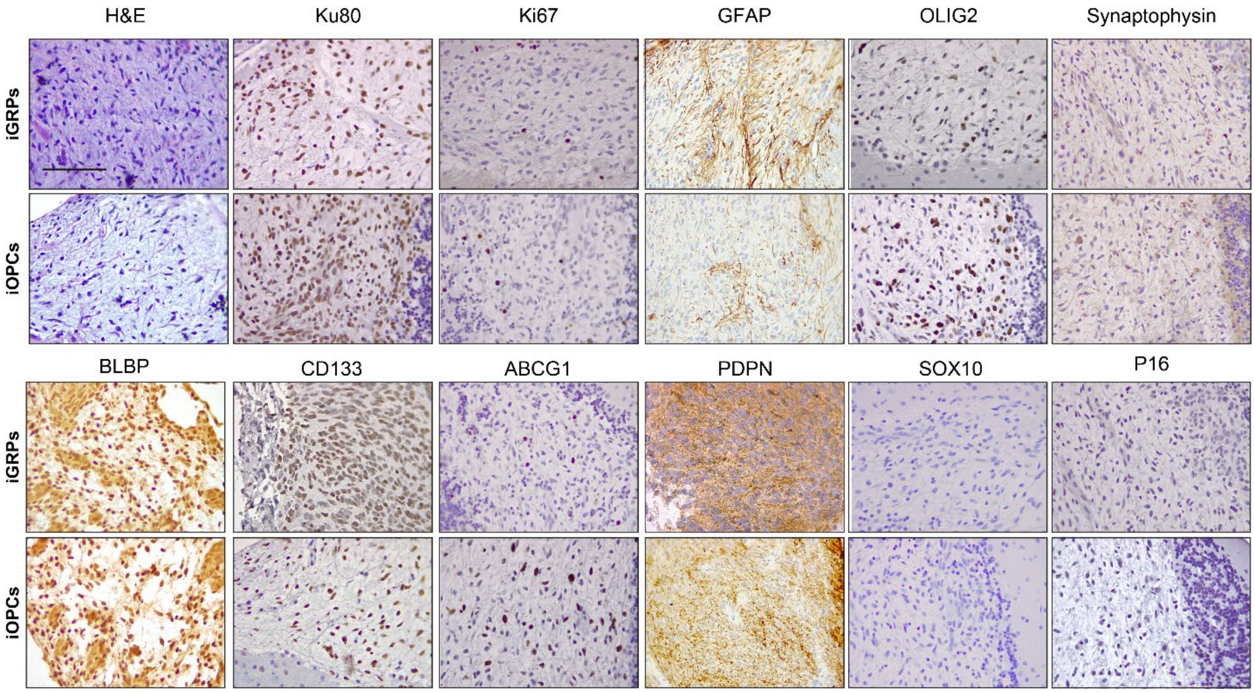

Additional File 1: Fig. S4 Immunostaining of *KIAA1549:BRAF*-expressing iGRP and iOPC LGGs

H&E, Ku80, Ki67, GFAP, OLIG2, synaptophysin, BLBP, CD133, ABCG1, PDPN, SOX10 and P16 immunostaining images of *KIAA1549:BRAF*-expressing iGRP- (top) and iOPC- (bottom) LGGs at 1mpi. Scale bar, 50µm.

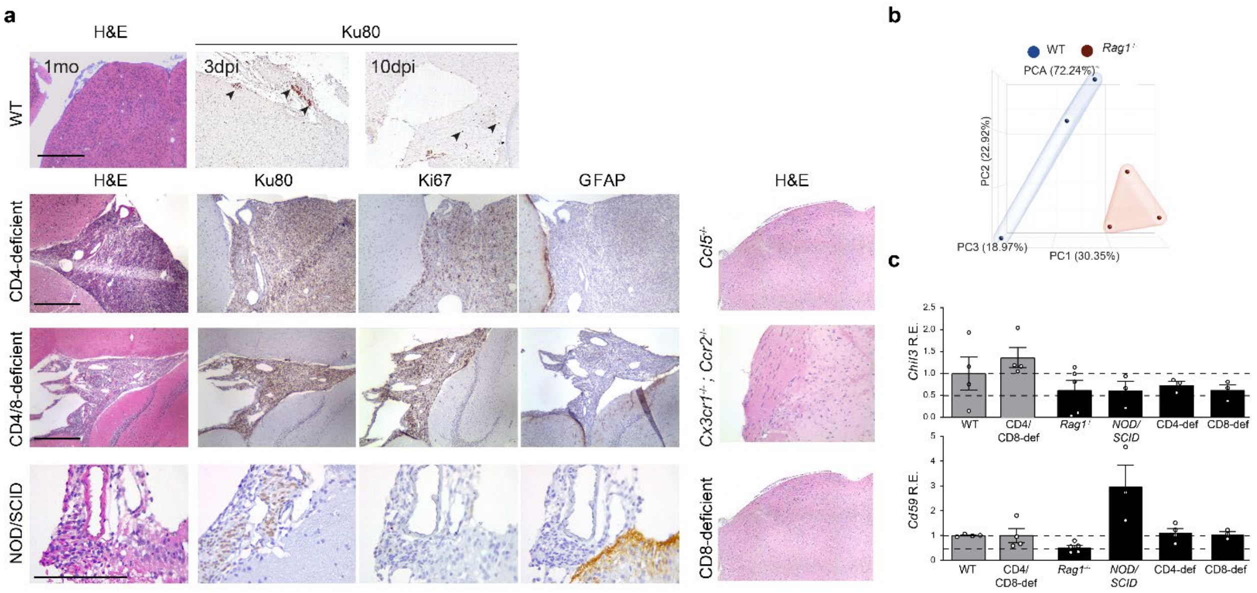

Additional File 1: Fig. S5 Analysis of iNPC-injected mice and RNA expression in different genetically engineered mouse strains (**a**) Representative images of H&E, CD3 (pan-T cell marker), Ku80, Ki67 and GFAP immunostaining of LGGs in *Rag1*$^{-/-}$, CD4-deficient, CD4/8-deficient, NOD/SCID mice, as well as H&E and CD3 staining of non-tumor-bearing wild type (WT), CD8-deficient, and *Cx3cr1*$^{-/-}$;*Ccr2*$^{-/-}$ mice one month after injection. Scale bars, 100µm. (**b**) PCA plot of RNA sequencing performed on the brainstems of naïve WT and *Rag1*$^{-/-}$ mice. (**c**) Relative expression (R.E.) of *Chil3* and *Cd59* in WT, CD8-deficient, *Rag1*$^{-/-}$, NOD/SCID, CD4/8-deficient, and CD4-deficient brainstem samples. Transcript expression is normalized to *Gapdh* expression. CTL, CD4/8-deficient mice, n=4; *Rag1*$^{-/-}$ mice, n=5; NOD/SCID, CD4-deficient, CD8-deficient mice, each n=3 independently-generated samples. Data are represented as means ± SEM. Dashed lines indicate 0.5- and 1-fold relative expression.

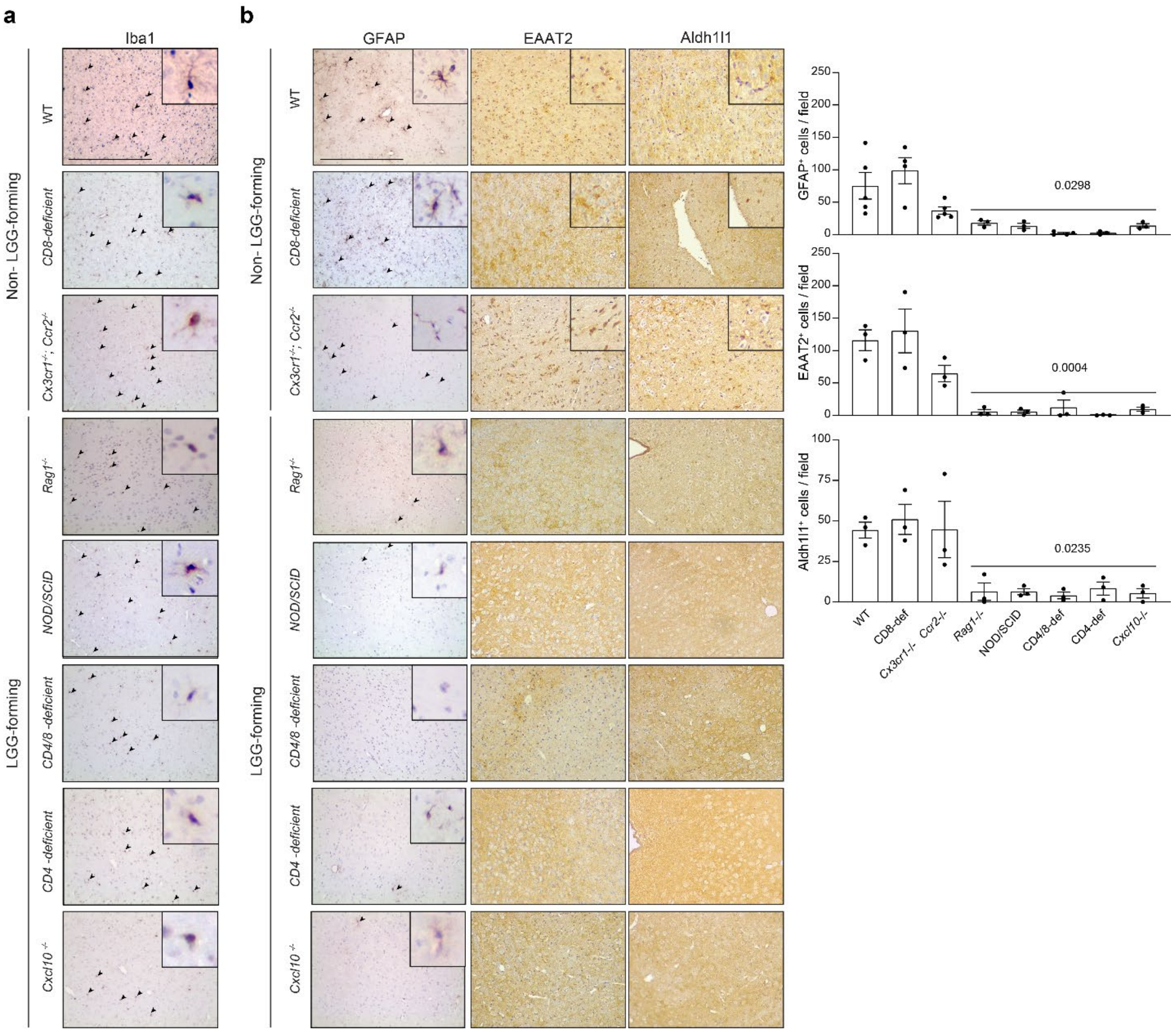

Additional File 1: Fig. S6 Immunostaining of naïve mouse brainstems (**a-b**) Immunostaining of naïve (uninjected) mouse brainstems reveals (**a**) unaltered microglial (Iba1^{+}) content, but (**b**) reduced GFAP^{+} , EAAT2^{+} and Aldh1l1^{+} cells in mouse strains permissive of LGG formation (*Rag1*$^{-/-}$, NOD/SCID, CD4/8-deficient, CD4-deficient, and *Cxcl10*$^{-/-}$ mice) relative to those that do not form LGGs (WT, CD8-deficient, and *Cx3cr1*$^{-/-}$;*Ccr2*$^{-/-}$ mice). Data are represented as means ± SEM. One-way ANOVA with Bonferroni post-test correction. Individual p values are indicated within each graph. Scale bars, 100μm.

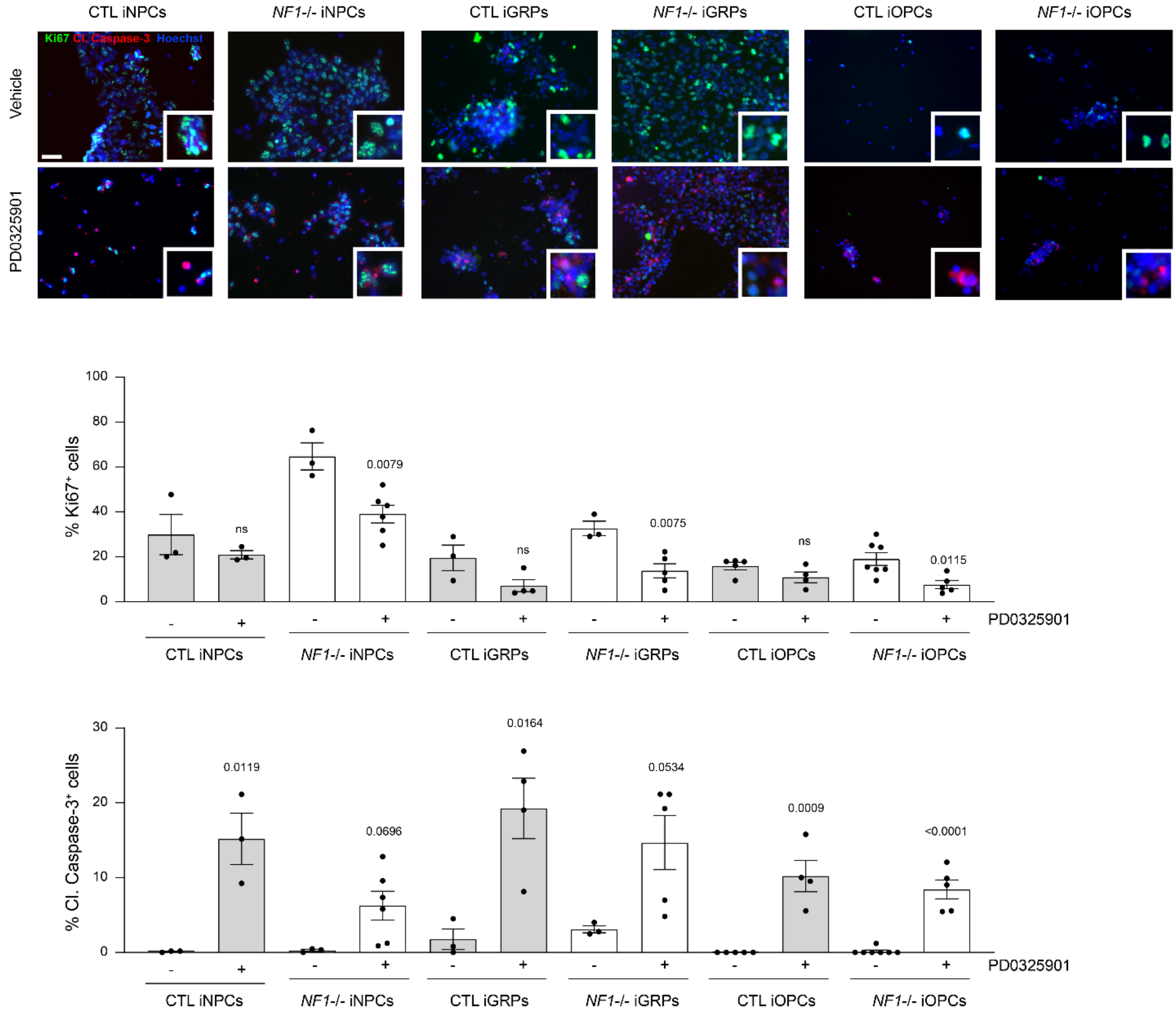

Additional File 1: Fig. S7 In vitro treatment of iNPCs, iGRPs and iOPCs with PD0325901. PD0325901 treatment of isogenic control (CTL) or *NF1*-null iNPCs, iGRPs and iOPCs decreases proliferation (Ki67) and increases apoptosis (cleaved caspase-3). Data are represented as means ± SEM. 2-tailed student's t-test. Individual p values are indicated within each graph. ns, not significant. Scale bars, 100µm.

a

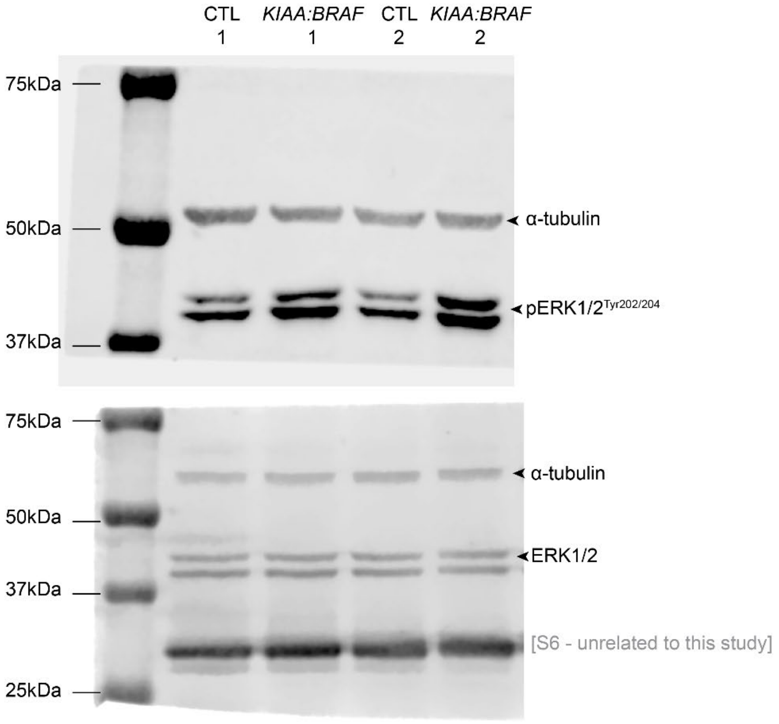

b

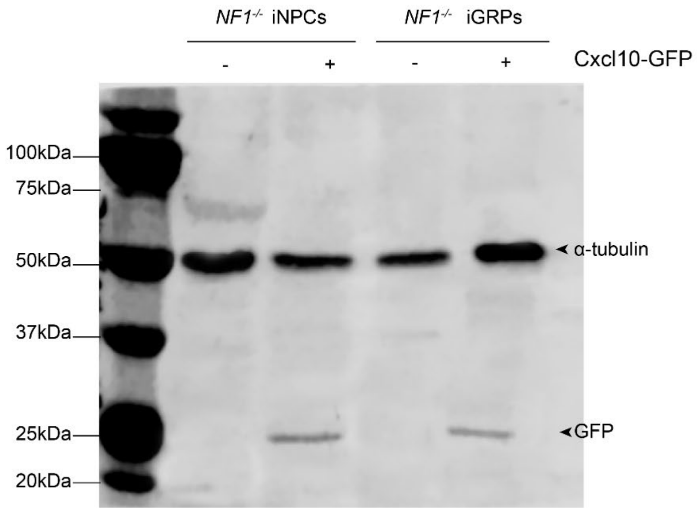

Additional File 1: Fig. S8 Uncropped western blot images (**a**) Uncropped immunoblot demonstrating increased phospho-ERK1/2$^{Tyr202/204}$ relative to total ERK1/2 in *KIAA1549:BRAF*-iNPCs and isogenic controls (CTLs). (**b**) Uncropped immunoblot demonstrating ectopic expression of murine Cxcl10 in *2041C*>T$^{-/-}$ iNPCs and iGRPs (GFP expression).
